# Evolution of One Species Increases Resistance to Invasion in a Simple Synthetic Community

**DOI:** 10.1007/s00248-025-02618-w

**Published:** 2025-10-20

**Authors:** Storme Z. de Scally, Michael J. McDonald

**Affiliations:** 1 School of Biological Sciences, Monash University, Clayton, VIC Australia; 2 Centre to Impact AMR, Monash University, Clayton, VIC Australia; 3 ARC Centre of Excellence Centre for Mathematical Analysis of Cellular Systems, Melbourne, Australia

**Keywords:** Microbial community, Microbial evolution, Experimental evolution

## Abstract

The species that make up a microbial community determine its potential function. A major goal of microbial ecology is to make assemblages of microbes — synthetic communities — with targeted applications. Replacing a dysfunctional community with a synthetic microbial community can have transformative impacts upon a host or ecosystem, yet the introduced community may be outcompeted by local species or communities, resulting in transient effects. Here, we study a simple synthetic community comprised of two species — *E. coli* and *S. cerevisiae* — that have coevolved for either 0, 1000 or 4000 generations, and evaluate the potential for 12 bacterial strains, from five species, to invade. We find that the dominant species (*E. coli*) in the community protects the less dominant species from being outcompeted during an invasion, and that this effect is strengthened by longer periods of coevolution. Using a mathematical model, we show how prolonged co-evolution leads to protective effects for a community member sensitive to displacement.

**Supplementary Information:**

The online version contains supplementary material available at 10.1007/s00248-025-02618-w.

## Introduction

Natural microbial communities can have a large effect on host and ecosystem function [20, 22, 48] so synthetic microbial communities that maximise the positive impacts are an attractive proposition [17, 42, 44]. Previous work has shown that a synthetic community’s composition can be tuned towards a specific outcome, either by curating a set of individually characterised microbes, or enriching a beneficial subset from within an existing community [44]. One approach to improve the performance of synthetic microbial communities is by artificial selection [3, 41, 47] — passaging the community under selection for a desired function [39]. These studies have shown that selection on microbial community composition can result in a microbial community that can have a reproducible impact in diverse contexts [1, 24, 32, 35]. However, in some cases, it is unclear whether experiments selecting for community-level traits improve function by ecological sorting — where species are discarded or retained, or by the genetic evolution of individual species’ populations within the community.

A major barrier to the application of synthetic microbial communities is their failure to establish, and stably modify, ecosystem function [2, 26]. Choosing the species for a synthetic community based on function may come at the cost of discarding species that are important for ecological stability [21]. This is important because, for many applications, a synthetic community will encounter an already established group of microbes. Microbes that are better adapted may be able to displace members of the synthetic community, driving changes in community function. The long-term stability of a desired function may require that a synthetic community has been selected for ecological and evolutionary stability, as well as for a desired functional output.

There are a growing number of evolution experiments that have tracked the genetic evolution of individual species in the context of a microbial community. The presence of a community tends to alter the outcomes of evolution [4, 29], although see [34]. In some cases, when a microbial community is selected to maintain its composition or function, the evolution of individual species is restricted to changes that maintain, or even potentially improve, the performance of the community [5, 10]. In contrast, if there is no shared evolutionary history, or no community to enforce constraints on the evolution of individual species, an evolving population can rapidly evolve traits that are not conducive to community stability [5, 16, 40]. Such an evolved type is unable to stably coexist within the community, which may ultimately disrupt function.

These results from the experimental evolution of microbial communities have implications for synthetic communities. Generating a synthetic community that can resist displacement by microbes outside the community is challenging because each of the community’s member-species may be vulnerable to different invader species [42]. In addition, a desirable function of a member species may come at the cost of the capacity to compete with an invader species. For example, if a desired species produces a public good, such as a secreted protein, this could come with a growth cost that makes this strain vulnerable to invasion by a strain that inhabits a similar niche, but does not produce the public good [37]. Several studies have addressed the difficulty of establishing beneficial communities in natural environments, and the importance of local adaptation to microbial community assembly and persistence [6, 14, 15].

In a previous work, we evolved *E. coli* MG1655 and *S. cerevisiae* R1158 in co-culture for 4000 generations, showing that both species evolved genetic changes that caused ecological stability — the capacity for both species to invade the other from low frequencies and converge on a stable, two-species equilibrium [4, 7]. In this study, our goal is to determine whether extended periods of evolution and adaptation would change the susceptibility of this simple two-species community to invasion by other microbial species.

## Methods

### Strains

The co-evolved *E. coli*-yeast pairs used this study were derived from *E. coli* MG1655 K-12 F^–^ λ^–^ *ilvG*^–^ *rfb-50 rph-1* and the haploid, non-recombining *S. cerevisiae* strain R1158 *trp1::Hph URA::CMV-tTA MATa his3-1 leu2-0 met15-0*. Versions of these two strains used in this study were the ancestral, 1000 [4] and 4000 [7] generation evolved cultures. The competitor strains were *Pseudomonas fluorescens SBW25*, *Pseudomonas aeruginosa K112 (ATCC 19429)*, *Staphylococcus aureus A134 ATCC 6538*, *Escherichia coli* (EPEC serotype 0127:H6, EAEC042, top10, MG1655-*ΔfhuA*::*bla* and WT MG1655) and *A. baumannii* (strains AB900, A9844, ATCC 19606 T, ATCC 17978 and AB5075).

### Phylogenetics and DNA Sequencing

Phylogenetic similarity between strains was assessed using multiple sequence alignment of the 16S rRNA gene. 16S rRNA sequences from each bacterial species were downloaded from NCBI and combined into a single multiple sequence alignment FASTA file which was then uploaded to CLUSTALW. The subsequent sequence alignment and phylogenetic analysis were performed using the “build” function of ETE3 3.1.2 [23]. RAxML v8.2.11 was used to infer a maximum likelihood tree, using the model “GTRGAMMA” with other parameters set to default [46]. Support for tree branches were computed out of 100 bootstrapped trees.

To verify that the genome sequences of our 4000-generation and 1000-generation clones were identical to the clones taken from the population and used in previous studies, we sequenced the exact clones used in this study. Genomic DNA was extracted from each sample using the GenElute™ Bacterial Genomic DNA Kit (Sigma-Aldrich, NA2110). The extracted DNA was sent to Azenta (Suzhou, China) for library preparation and sequencing on the Illumina NovaSeq 6000 platform. The sequencing reads provided by Azenta were pre-trimmed for adapters. Additional read filtering and trimming were performed using the BBDuk package (http://jgi.doe.gov/data-and-tools/bbtools/) with default parameters. *E. coli* samples were aligned to the *E. coli* MG1655 reference genome (EC_ANC, Accession: SAMN16401120, Bioproject: PRJNA668197), and yeast samples were aligned to the *S. cerevisiae* R1158 reference genome (SC_ANC, Accession: SAMN16401106, Bioproject: PRJNA668197). Genetic variants were identified using the breseq pipeline. The raw data for the 1000-generation populations (*E. coli* generation 4000*-clone2*, Accession: Bioproject: PRJNA1023613; *S. cerevisiae* generation 4000*-clone2*, Accession: Bioproject: PRJNA1023613) were included in the analysis.

### Growth Media

All strains were grown in high glucose medium “High Glucose Medium” (HGM) [51]) (13.3 g/L KH_2_PO_4_, 4 g/L (NH_4_)_2_HPO_4_, 1.7 g/L citric acid, 0.0084 g/L EDTA, 0.0025 g/L CoCl_2_, 0.015 g/L MnCl_2_, 0.0015 g/L CuCl_2_, 0.003 g/L H_3_BO_3_, 0.0025 g/L Na_2_MoO_4_, 0.008 g/L Zn(CH_3_COO)_2_), 0.06 g/L Fe(III) citrate, 0.0045 g/L thiamine, 1.3 g/L MgSO4, pH 7.0, containing 5 g/L yeast extract and 40 g/L glucose). When plating on solid media we used Yeast extract Peptone Dextrose (YPD) Agar. For selective plating, we supplemented media with either 1 mg/mL cycloheximide to select for *E. coli,* or 1 mg/mL tetracycline to select for yeast. Cultures were grown at 28 °C shaken during the competition experiments and to determine growth dynamics in monoculture. To check for the presence of competitor strains we plated onto Lysogeny Broth (LB) agar plates supplemented with antibiotics.

### Measurement of Growth Dynamics

Single colonies of each strain were picked from either LB (all bacterial strains) or YPD (*S. cerevisiae*) agar plates and grown in 10 mL HGM overnight at 28 °C shaking. Optical density (OD_600_) was measured and standardised to 0.006 for prokaryotic strains and 0.01 for yeast. Samples were then placed onto a 96-well plate in a randomised design with a minimum of 5 replicates. Population growth was then monitored by tracking OD for 24 h, with results recorded every 10 min. All strains used in this study could reach carrying capacity in HGM within 24 h. Analyses of growth reader data was carried out using R studio, (*growthcurver*) [45].

### Competition Experiments

To initiate the competition experiments, single colonies of each strain were grown to saturation overnight in 3 mL of HGM in a 15 mL Falcon tube. Strains were then diluted 1:2^5^ in phosphate-buffered saline (PBS) and mixed in approximately equal cell ratios. Ratios were calculated after determining the relationship between colony forming units (CFUs) and optical density (OD) for each species. Overnight cultures were mixed at approximate cell densities of 1 × 10^8^ cells ml^−1^ for bacterial pairs and 1 × 10^7^ cells ml^−1^ when mixed in co-cultures with yeast. Replicate cultures were grown in individually packaged, sterile 96 well round bottom plates for 24 h, shaking at 700 rpm. For 7 daily cycles (70 generations) of growth and dilution, cultures were diluted 1:2^5^ (4 μL into 124 μL) into sterile PBS for flow cytometry and further diluted 1:2^5^ (4 μL into 124 μL) into fresh HGM. Following each transfer, samples were mixed with 75% glycerol and stored at −  80 °C for downstream analyses. Replicates for each treatment were distributed across different wells within 96-well plates and across multiple experimental days. This design ensures that the reported error bars incorporate both within-plate and between-plate variation. For three-way co-culture experiments, *E. coli* and yeast cells were initially mixed as described above and subjected to three daily cycles of growth and dilution, allowing *E. coli*-yeast pairs to reach equilibrium. At this point, a competitor bacterial species was introduced into the co-culture at approximately equal cell ratios, creating a three-species microbial community. Two- and three-way competitions were carried out with five replicates, and the outcome designated as coexistence or extirpation based on the majority, for example, if three replicates coexist, then the outcome was designated “coexist”.

### Flow Cytometry and Plating to Determine Relative Population Sizes

Where cells could be distinguished by size, cell counts were measured by flow cytometry on an LSR Fortessa X20c to a maximum of 100,000 events. Gates were established using diluted overnight cultures of yeast and *E. coli* ancestral and evolved strains. Cell size was observed using forward and side scatter parameters and gates were drawn for each species after a maximum of 100,000 events. The forward scatter value chosen to observe both yeast and *E. coli* cells was 350, side scatter was 180 and the threshold was set to 500. As *E. coli* cells are smaller than yeast, *E. coli* was gated to the left of the forward scatter, side scatter plot, whereas yeast was gated to the right of the plot. For each plot, the forward scatter area was plotted on the x-axis and side scatter area on the y-axis. After initial gating on the LSR Fortessa X20c, gates were reconfirmed using Floreada.io software after downloading the raw FACS data files from the server (Fig. S4). Detected events were then assigned to either the yeast or *E. coli* gate, and the number of events or counts were converted to relative frequency for each species. Flow cytometry rapidly distinguishes *E. coli* and yeast populations in co-culture based on size and fluorescence properties. Although it measures relative rather than absolute population sizes, it reliably tracks shifts in equilibrium frequencies over time or after perturbations. While this approach is sufficient for monitoring evolutionary and ecological trends, it cannot reliably indicate when a strain has become extirpated from a sample. In co-cultures containing multiple bacterial species, cell populations could not be distinguished by size alone, so colony-forming units (CFUs) were counted using selective plating. Selective plating was performed either with antibiotics or for *S. aureus*, based on distinguishable colony morphologies. Antibiotic resistance profiles for each bacterial strain were initially obtained from the literature and subsequently confirmed by plating strains on LB agar with a range of antibiotic concentrations, both above and below the reported minimum inhibitory concentration (MIC). Specifically, chloramphenicol (100 µg/mL) was used to select for all tested *A. baumannii* strains, ampicillin (32 µg/mL) for *P. aeruginosa*, *P. fluorescens*, *E. coli* EPEC, and *E. coli* MG1655 *bla*, and streptomycin (100 µg/mL) for *E. coli* Top10 and *E. coli* EAEC042. *S. aureus* was distinguished from *E. coli* MG1655 based on distinct colony morphologies. Yeast could be selected for by plating on YPD plates containing ampicillin 100 µg/mL and tetracycline (25 µg/mL). Plating was done by spotting 20 μL of co-culture onto selection plates, and the number of colonies counted. The use of a selectable marker enabled an extremely low limit of detection, and we would expect to be able to detect cells until the subpopulation size had fallen below 10 cells, out of a total population of millions. In the case of *S. aureus*, since we didn’t have a selectable marker, we could only screen 100’s of colonies, so our limit of detection was 1–0.1%.

### Statistical Analyses

All statistical analyses were performed using RStudio (R version 4.4.2). Paired *t*-tests and Bonferroni corrections were performed using the t.test and p.adjust functions in R baseplot. Correlation of growth rate and final frequency for yeast and *E. coli* ancestral, generation 1000 and generation 4000 strains was performed in RStudio. Data were checked for normality using qqplots, histograms and the Shapiro-Wilk test in RStudio. Spearman’s rank correlation test was also performed on all samples, because while growth rate was normally distributed, final frequency was non-parametric. No significant correlation was found.

### Spent Media Assays

Spent media assays were performed to analyse pairwise negative interactions and niche overlap between strains. Spent media was generated by inoculating a single colony of each strain (*n* = 12) into 3 mL of HGM medium and growing the cultures at 28 °C with shaking for 24 h. Following incubation, the cultures were centrifuged at 4000 × g for 4 min, or for additional spins as necessary, until all cells were pelleted and the liquid medium was clear. The supernatant was then filter-sterilized using a 0.2 μm filter to produce the spent media.

For each assay, spent media from *E. coli* strains (ancestral, G1000, G4000; *n* = 3) or yeast strains (ancestral, G1000, G4000; *n* = 3) was used to grow each of the 12 strains. Reciprocally, all *E. coli* and yeast generations (*n* = 6) were grown in the spent media of each competitor strain (*n* = 12). To prepare cells for growth in spent media, the cultures were centrifuged as described above, and the supernatant was discarded. PBS was added to the cell pellet at a 1:1 ratio to the original culture volume to control for cell density. The resuspended cells were centrifuged again at 4000 × g for 4 min, and the supernatant was removed. The final cell pellets were resuspended in an equal volume of PBS. A 4-μL aliquot of the washed and resuspended cells was added to 128 μL of spent media in a 96-well plate, with 5 replicates per strain and per spent media type. For example, to assess the growth of competitors in G1000 *E. coli* spent media, 4 μL of each strain was added to 128 μL of G1000 *E. coli* spent media in a randomized 96-well plate design (*n* = 5 replicates × 12 strains = 60 wells). Population growth was monitored by tracking optical density at 600 nm (OD_600_) for 24 h, with measurements taken every 10 min. All assays were conducted at 28 °C with shaking. Growth curve analyses were performed in R using the package *growthcurver* [45].

### Prediction of Interaction Outcomes using Growth Rate

We used growth rates to make predictions about the outcome of two-way competitions (Fig. 3D**,** Supplementary Methods). If a bacterial competitor strain had a mean growth rate that was greater than 1 standard deviation above the *E. coli* or yeast strain that it was competing with, we predicted the competitor would drive the *E. coli* or yeast strain extinct. Conversely, if the growth rates were within 1 standard deviation, we predicted that these strains could coexist (Supplementary Methods).

### Simulated Interaction Values and Competition Outcomes

To better understand the role of evolution on species interactions and community assembly, we simulated competition outcomes of a three-species community (Supplementary Methods). We modified the classic predator–prey Lotka-Volterra competition model to include frequency dependence and ran simulations with varied parameter values to model three-species competition outcomes. Below are the three-species models without (Eqs. (1.1), (1.2), (1.3)) and with (Eqs. (2.1), (2.2), (2.3)) frequency dependence. We used the models with frequency dependence to simulate our experimental observations. Solving the equations show how the population size of each species changes over time, depending on other parameter values within the equation. All simulations using ordinary differential equations were run using RStudio. Subscripts B, Y and E refer to the three species in our trio competitions, i.e., competitor bacteria (B), the yeast *S. cerevisiae* (Y) and *E. coli* MG1655 (E). The initial population size of a species is denoted by N. For example, N_E_ is the starting population size of *E. coli*. The intrinsic growth rate is indicated as r, e.g. r_B_ is the intrinsic growth rate of the competitor bacteria. K is the carrying capacity of a species, e.g. K_Y_ is the carrying capacity of yeast. The interaction coefficient between two species is alpha (α), where a_ij_ is the effect of species j on species i and a_ji_ is the effect of species i on species j. For example, α_EY_ is the effect of yeast on *E. coli*, whereas α_YE_ is the effect of *E. coli* on yeast.

1.1 $$\frac{d{N}_{E}}{dt}={N}_{E}{r}_{E}\left(1-\frac{{N}_{E}+{\alpha }_{EY}{N}_{Y }+ {\alpha }_{EB}{N}_{B }}{{K}_{E}}\right)$$

1.2 $$\frac{d{N}_{Y}}{dt}={N}_{Y}{r}_{Y}\left(1-\frac{{N}_{Y}+{\alpha }_{YB}{N}_{B }+ {\alpha }_{YE}{N}_{E }}{{K}_{Y}}\right)$$

1.3 $$\frac{d{N}_{B}}{dt}={N}_{B}{r}_{B}\left(1-\frac{{N}_{B}+{\alpha }_{BY}{N}_{Y }+ {\alpha }_{BE}{N}_{E }}{{K}_{B}}\right)$$

2.1 $$\frac{d{N}_{E}}{dt}={N}_{E}{r}_{E}\left(1-\frac{{N}_{E}+{\alpha }_{EY}{{(\frac{{N}_{Y }}{{{{{N}_{Y }+}N}_{B }+N}_{E }})}N}_{Y }+ {\alpha }_{EB}{{(\frac{{N}_{B }}{{{{{N}_{Y }+}N}_{B }+N}_{E }})}N}_{B }}{{K}_{E}}\right)$$

2.2 $$\frac{d{N}_{Y}}{dt}={N}_{Y}{r}_{Y}\left(1-\frac{{N}_{Y}+{\alpha }_{YB}{(\frac{{N}_{B }}{{{{{N}_{Y }+}N}_{B }+N}_{E }})N}_{B }+ {\alpha }_{YE}{(\frac{{N}_{E }}{{{{{N}_{Y }+}N}_{B }+N}_{E }})N}_{E }}{{K}_{Y}}\right)$$

2.3 $$\frac{d{N}_{B}}{dt}={N}_{B}{r}_{B}\left(1-\frac{{N}_{B}+{\alpha }_{BY}{(\frac{{N}_{Y }}{{{{{N}_{Y }+}N}_{B }+N}_{E }})N}_{Y }+ {\alpha }_{BE}{(\frac{{N}_{E }}{{{{{N}_{Y }+}N}_{B }+N}_{E }})N}_{E }}{{K}_{B}}\right)$$

We first assessed whether the frequency-dependent LV equations could generate the competition outcomes we observed empirically with the parameter values observed during the experiment. We measured the intrinsic growth rate of each strain as previously described above and determined each strain’s carrying capacity after 24 h of growth in the experimental HGM media.

Following [7], we found that the modified model matched empirical observations of stable, two-species *E. coli*-yeast coculture. Next we incorporated the third species and explored how altering the interaction values, alpha, resulted in different outcomes for three way co-culture. Specifically, we aimed to determine the range of alpha values which would result in coexistence or exclusion of one or more species. Using the frequency dependent LV Eqs. (2.1), (2.2) and (2.3), we simulated the population size of each species after 24 h for a range of alpha values (0–3 in steps of 0.01) and plotted the range of alphas that resulted in either coexistence or exclusion. For simulations of each alpha pair, all other alpha values were kept at a low, constant value of 0.01, indicating the species were not interacting. For example, when assessing alpha.be and alpha.eb, the other alpha values alpha.yb, alpha.by, alpha.ey and alpha.ye were all 0.01. Starting population sizes and carrying capacities were parameterised based on the experiment. Data shown in Fig. 4 is based on empirical measures of yeast, *E. coli* and *A. baumannii* AB19606T. However, altering values to match each species exact measurements does not change the relationship between alpha values plotted, and rather represents a general representation of three species interactions. Populations were counted as extinct (Fig. 4, purple outcomes) if the population size was 10,000 or lower; this threshold was determined as cultures in our experiment were diluted 1000 × daily, which would reduce a population size of 10^4^ cells down to 10^2^. As an alpha value of 1 or greater reduces cell numbers tenfold or more, a reduction in population size to 10,000 after 24 h would lead to extinction after 48 h, i.e. 10,000 reduced to 100 after 24 h, further reduced to 0 after an additional 24 h. All simulations were run in RStudio using the base R package, and simulation outcomes were plotted using *ggplot2* [50].

## Results

### *E. coli *and Yeast Form an Ecologically Stable, Two-Species Community

**Fig. 1:**
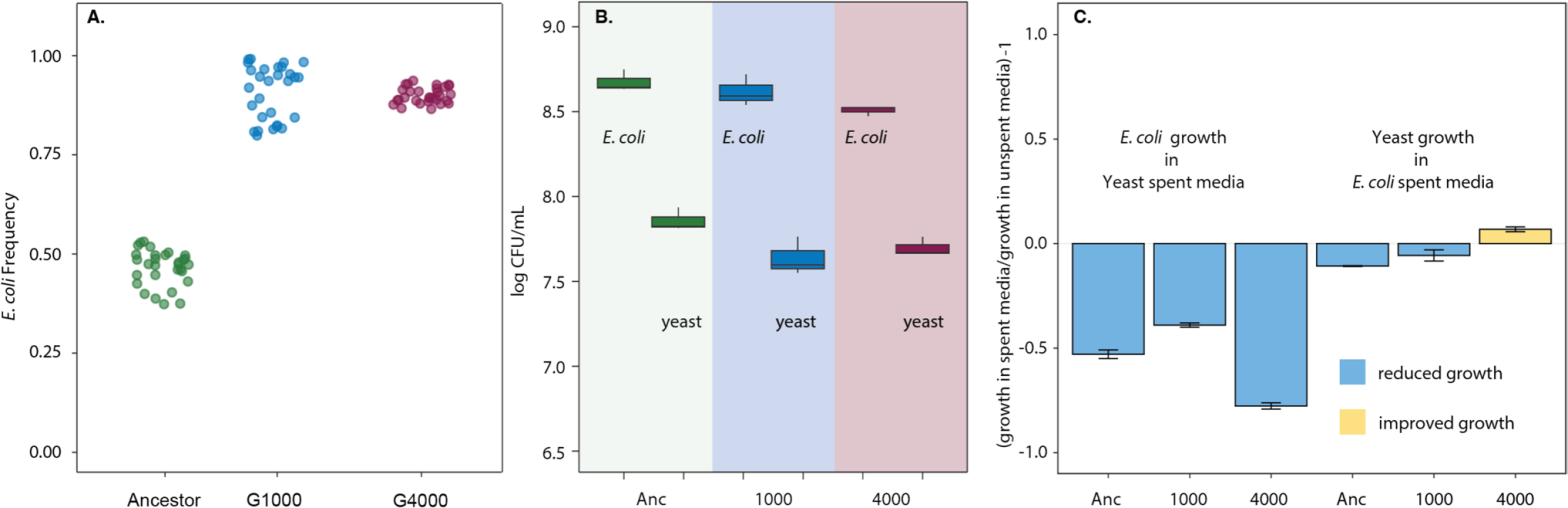
Co-evolved *E. coli*-yeast pairs. (**A**) Equilibrium frequencies for *E. coli*-yeast combinations that were co-cultured for either 0 generations, i.e. the ancestor (green), 1000 generations (blue) or 4000 generations (red). Each point represents an independent co-culture. (**B**) Population size at equilibrium, for *E. coli* and yeast in co-culture. (**C**) Maximum carrying capacity (Max OD, or stationary phase density) of *E. coli* and yeast in the spent media of their “partner” strain from the same generation. Y axis indicated growth in spent media divided by growth in unspent media, minus 1. For example, the rightmost column shows that 4000 generation evolved yeast grows significantly better in media that has already been spent by 4000 generation *E. coli*, than ancestral yeast grows in ancestral *E. coli* spent media (paired *t*-test *t* =  − 14.35, *p* = 1.37 × 10^−4^, *d.f.* = 4). Error bars indicate standard error of the mean

We previously evolved co-cultured populations of *E. coli* MG1655 and *S. cerevisiae* (hereafter referred to as yeast) for 4000 generations of laboratory evolution. Over this period, *E. coli*-yeast pairs evolved the capacity to quickly reach a stable equilibrium with both *E. coli* and yeast coexisting, even when cultures were initiated from a broad range of starting *E.coli*/yeast ratios [4, 7]. Importantly, stability was contingent on evolution in both species — combining ancestor yeast or *E. coli* with an evolved *E. coli* or yeast partner disrupted coexistence [4]. This ecologically stable two-species system provides the opportunity to study the role of evolution on community-level traits, such as resistance of the two-species community to invasion by a third species. In this study, we focus on three *E. coli*-yeast pairs, taken from different time points during the evolution experiment: The ancestral generation (G0), generation 1000 (G1000), and generation 4000 (G4000) [7]. We used flow cytometry to evaluate the equilibrium state of the three pairs (Supplementary Methods), finding variation in the relative frequency of *E. coli* and yeast in the ancestral *E. coli-*yeast pair (~ 35–55% *E. coli*), the 1000 generation *E. coli-*yeast pair (80–98% *E. coli*)*,* and the 4000 generation *E. coli-*yeast pair (85–95% *E. coli*, Fig. 1A). In each instance, we express the equilibrium frequency as the frequency of *E. coli*, so that an equilibrium frequency of 85% means that 15% of the cells sorted by flow cytometry in the co-culture are yeast. Equilibrium frequencies are reached within 1–2 days. In addition to flow cytometry, which can provide information about the relative proportion of cell types, we determined the absolute population sizes of *E. coli* and yeast in co-culture, establishing that in co-cultures there is typically at least 10 × as many *E. coli* cells as yeast cells (Supplementary Methods, Fig. 1B).

### Yeast Evolves to Utilise Different Resources to E. coli in Co-culture

To determine how the resource needs of *E. coli* and yeast compare in a two-species community, we performed growth assays using both fresh media and media previously consumed by the other species (see Supplementary Methods). Spent media assays offer a way to quantify the extent of resource-use overlap between species: If a species grows just as well in spent media as in fresh media, it suggests that its resource requirements do not overlap significantly with those of the other species. We found that G4000 yeast evolved to perform just as well in G4000 *E. coli’s* spent media as in fresh media, suggesting that yeast had evolved to use different resources than *E. coli* (Fig. 1C, paired *t*-tests with Bonferroni correction between spent and fresh media for each generation: ancestral yeast in ancestral *E. coli* spent media, *p* = 2.05 × 10^−4^, *df* = 4, *t* = 17.09; 1000-generation evolved yeast in 1000-generation evolved *E. coli* spent media, *p* = 0.58, *df* = 4, *t* = 1.556; 4000-generation evolved yeast in 4000-generation evolved *E. coli* spent media, *p* = 0.06, df = 4, *t* = −3.73). We found that all generations of *E. coli* grew more poorly in yeast-spent media than in unspent media, suggesting either that in the absence of *E. coli,* yeast consumes different resources than when in co-culture with *E. coli*, or that yeast produces a substance that inhibits the growth of *E. coli* (Fig. 1C, paired *t*-tests with Bonferroni correction between spent and fresh media for each generation: ancestral *E. coli* in ancestral yeast spent media, *p* = 7.6 × 10^−4^, *d.f* = 4, *t* = 12.26; 1000-generation evolved *E. coli* in 1000-generation evolved yeast spent media, *p* = 9.03 × 10^−5^, *df* = 4, *t* = 21.05; 4000-generation evolved yeast in 4000-generation evolved *E. coli* spent media, *p* = 6.72 × 10^−5^, *d.f* = 4, *t* = 22.677).

### *E. coli*, but not Yeast, Evolves Greater Resistance to Invasion from Competitor Bacteria Species

**Fig. 2:**
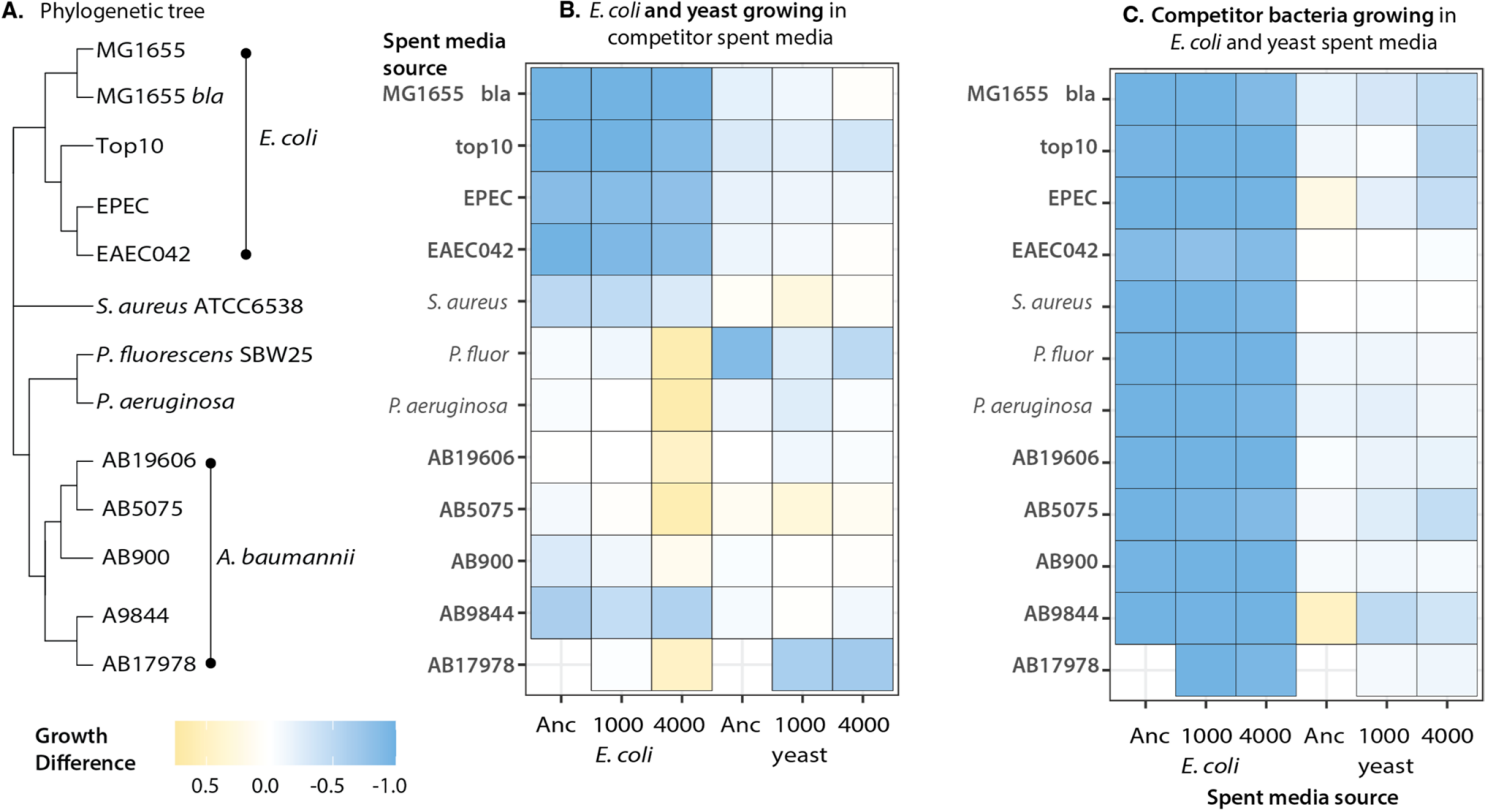
Competitor strains spent media assays and pairwise growth assay outcomes. (**A**) Phylogenetic relationship of bacterial strains used in this study. Branch lengths do not indicate genetic distance. (**B**) Growth of *E. coli* and yeast in the spent media of the invading bacterial strains. *E. coli* showed varied growth in the spent media of “competitor” strains; while ancestral and 1000 generation *E. coli* grew poorly (dark blue), or showed intermediate growth (light shading) in the spent media of several “competitor” strains, 4000-generation *E. coli* showed improved growth (yellow) in *Pseudomonas* and some *Acinetobacter* spent media. In contrast, yeast showed intermediate (light shading) to improved growth (yellow) in the growth media spent by the competitor bacteria, with only a single case of poor growth (dark blue) in *P. fluorescens* spent media. (**C**) All competitor strains grew very poorly in *E. coli* spent media, while species and strains showed intermediate and some improved growth, in yeast spent media. Growth difference was determined by dividing growth in spent media by growth in unspent media, and subtracting 1

Next, we tested whether *E. coli*-yeast pairs that had evolved together for more generations were better able to resist invasion by a third species. We selected five bacterial species — *Escherichia coli*, *Staphylococcus aureus*, *Pseudomonas fluorescens*, *Pseudomonas aeruginosa* and *Acinetobacter baumannii* (Fig. 2A, Table 1), pathogens with high invasiveness, colonization efficiency and environmental versatility [9, 27, 31, 43]. To test the importance of within-species variation, we selected four additional strains within the species *E. coli* and five strains of *A. baumannii*. Each of these 12 selected bacteria could attain high densities in HGM media (Fig. S2). Spent media assays indicated that the competitor species performed poorly in *E. coli* spent media but had a range of growth outcomes in yeast spent media (Fig. 2BC, Fig. S1, Supplementary Methods).

**Table 1:** Identity of species and strains used in the experimental procedure

Species	Strain	Sample ID	Lab ID
*Escherichia coli*	Ampicillin resistant MG1655 by *fhuA* knockout	MG1655 *bla*	MJM 490
*Escherichia coli*	MG1655 K-12 F^–^ λ^–^ *ilvG*^–^ *rfb-50 rph-1 ancestor*	EA	MJM 101
*Escherichia coli*	MG1655 K-12 F^–^ λ^–^ *ilvG*^–^ *rfb-50 rph-1 ancestor* G1000 evolved	E1	SDS 01
*Escherichia coli*	MG1655 K-12 F^–^ λ^–^ *ilvG*^–^ *rfb-50 rph-1 ancestor* G4000 evolved	E4	SDS 02
*Escherichia coli*	EAEC042	EAEC042	MJM 75
*Escherichia coli*	LA Top10	top10	MJM 80
*Escherichia coli*	EPEC serotype 0127:H6	EPEC	MJM 83
*Saccharomyces cerevisiae*	*R1158 trp1::Hph URA::CMV-tTA MATa his3-1 leu2-0 met15-0* ancestor	SA	SDS 03
*Saccharomyces cerevisiae*	*R1158 trp1::Hph URA::CMV-tTA MATa his3-1 leu2-0 met15-0* G1000 evolved	S1	SDS 04
*Saccharomyces cerevisiae*	*R1158 trp1::Hph URA::CMV-tTA MATa his3-1 leu2-0 met15-0* G4000 evolved	S4	SDS 05
*Acinetobacter baumannii*	ATCC 17978	AB17978	MJM 660
*Acinetobacter baumannii*	ATCC 19606T	AB19606T	MJM 661
*Acinetobacter baumannii*	AB5075	AB5075	MJM 569
*Acinetobacter baumannii*	A9844	AB9844	MJM 664
*Acinetobacter baumannii*	AB900	AB900	MJM 662
*Pseudomonas fluorescens*	K103	Pf	MJM 146
*Pseudomonas aeruginosa*	ATCC 19429	Pa	MJM 116
*Staphylococcus aureus*	ATCC 6538	Sa	MJM 115

**Fig. 3:**
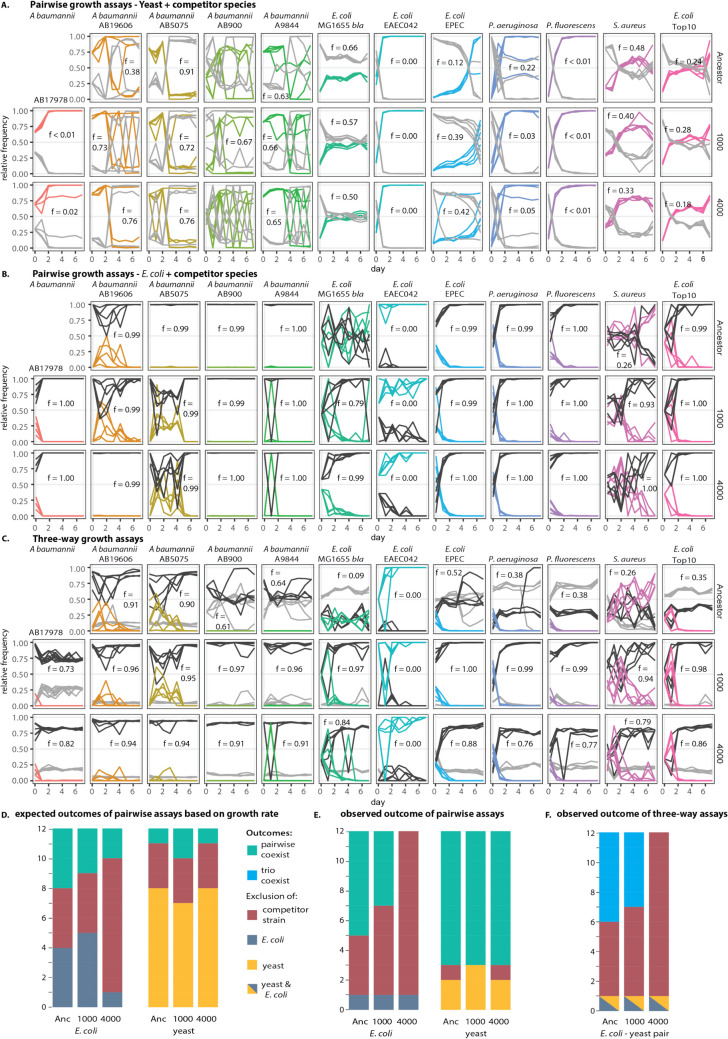
Three-way competitions are predicted by *E. coli* but not yeast pairwise assays. (**A**) Measurements of pairwise competitions between yeast (grey lines) and each competitor bacteria (coloured lines). The frequency of yeast at the final time point is indicated by inset text. (**B**) Measurements of pairwise competitions between E. coli (black lines) and each competitor bacteria (coloured lines). (**C**) Measurements of three-way competitions between yeast (grey lines), E. coli (black lines) and each competitor bacteria (coloured lines). The frequency of E. coli at the final time point is indicated by text. Each pair or set of lines indicates an independent 7-day experiment. Cultures for A. baumannii AB19978, ancestral S. cerevisiae, and ancestral E. coli failed to grow under the experimental conditions and are therefore not shown. (**D**) Predicted outcomes of pairwise competition assays, based on measurements of growth rates of each strain in monoculture. Growth rates of competitor bacteria were compared to growth rates of either E. coli or yeast (Fig. S2, Supplementary Methods). (**E**). Outcomes are either coexistence (green) or competitive exclusion of the competitor species (red), E. coli (blue) or yeast (yellow). For example, for (E), the three columns on left summarise the results of two-way assays between E. coli and the competitor strains and show that E. coli was excluded by only one of the competitor bacterial strains (E. coli EAEC042). (**F**) The three columns indicate 3-way assays between E. coli-yeast pairs and a competitor bacterial strain. The yellow-blue box indicates where both E. coli and yeast were excluded in the three-way growth assay. Two- and three-way competitions were carried out with five replicates, and the outcome designated as coexistence or extirpation based on the majority outcome, and extinctions confirmed with plate counts, where possible (Supplementary Methods)

We carried out pairwise co-cultures comprised of each of the “competitor” bacterial strains and either the G0 yeast ancestor, G1000 yeast or the G4000 yeast (Fig. 3A). And co-cultures of each of the “competitor” bacterial strains and either the G0 *E. coli* ancestor, G1000 *E. coli* or G4000 *E. coli* (Fig. 3B, Supplementary Methods). We tracked the frequency of each pairwise combination over 7 days, and report on the outcome of either exclusion or coexistence on the 7th day of co-culture. We define coexistence as the maintenance of both species for the 7-day experiment, where selective markers were, in most cases, able to allow for a low limit of detection (Supplementary Methods).

We found that ancestral and evolved yeast were not able to exclude any of the 12 introduced strains, although co-cultures of yeast and the introduced strain rarely resulted in the exclusion of yeast (Fig. 3A). Similarly, co-cultures of ancestral *E. coli* and the introduced strain more frequently resulted in coexistence than exclusion, where 8 out of 11 strains coexisted (Fig. 3B and E). In contrast, pairwise co-cultures of competitor bacteria with the evolved *E. coli* strains often resulted in exclusion of the competitor (9 out of 12; chi-square test, *X*^*2*^ = 5.47, *p* = 0.019). These results show that *E. coli* was better able to resist invasion as it became better adapted to the growth conditions of the experiment.

### Evolved *E. coli*-Yeast Pairs Exclude Competitors that can Outcompete Yeast

Next, we assessed the capacity of the competitor bacterial strains to coexist with the *E. coli*-yeast two species community, again tracking species frequencies over 7 days of co-culture (Fig. 3C). The ancestral *E. coli*-yeast pair coexisted with 5/11 of introduced competitor strains, the generation 1000 *E. coli*-yeast pair coexisted with 4/12 competitors and the G4000 *E. coli*-yeast pair excluded all but one of the competitors (*n* = 11/12 exclusions, Fig. 3C). In many cases, competitors were extirpated from the three-way co-cultures, although their transient presence could have a lasting effect on the composition of the communities that excluded them (Fig. 3C).

Consistent with previous studies [11, 33], we found that the growth of individual species in monoculture did not reliably predict the outcomes of two- or three-species co-cultures (Fig. 3D**, Fig. S3,** Supplementary Methods). Prior research has suggested that pairwise interactions are good predictors of three-species co-culture outcomes [8, 11, 33]. However, our pairwise interaction results (Fig. 3E) were inconsistent with 22% (8 out of 36) of the three-species co-cultures we observed (Fig. 3F). In each of these cases, the unexpected persistence of yeast in three-species cultures (Fig. 3C) caused the inconsistency.

This outcome is surprising because, in scenarios where *E. coli* > competitor bacteria > yeast, the expectation is that *E. coli* would outcompete both the introduced bacteria and the yeast [12, 19]. Yet, yeast persisted even as the introduced competitor was eliminated. For instance, G4000 *E. coli* outcompetes *P. aeruginosa* in pairwise culture, and *P. aeruginosa* outcompetes G4000 yeast in pairwise culture. Based on this, the expected outcome of three-species competition is the dominance of G4000 *E. coli*. Instead, we observed the coexistence of G4000 yeast and *E. coli*, with the extinction of *P. aeruginosa*. These results cannot be readily explained by the known population dynamics of the three species in pairwise cultures (Fig. 3D). They suggest that *E. coli* indirectly modifies the interaction due to the large negative effect on the competitor, enabling unexpected coexistence.

### *E. coli’s *Strong Interactions with Competitors Explain why Competitors Fail to Outcompete Yeast in Three-Species Co-cultures

**Fig. 4:**
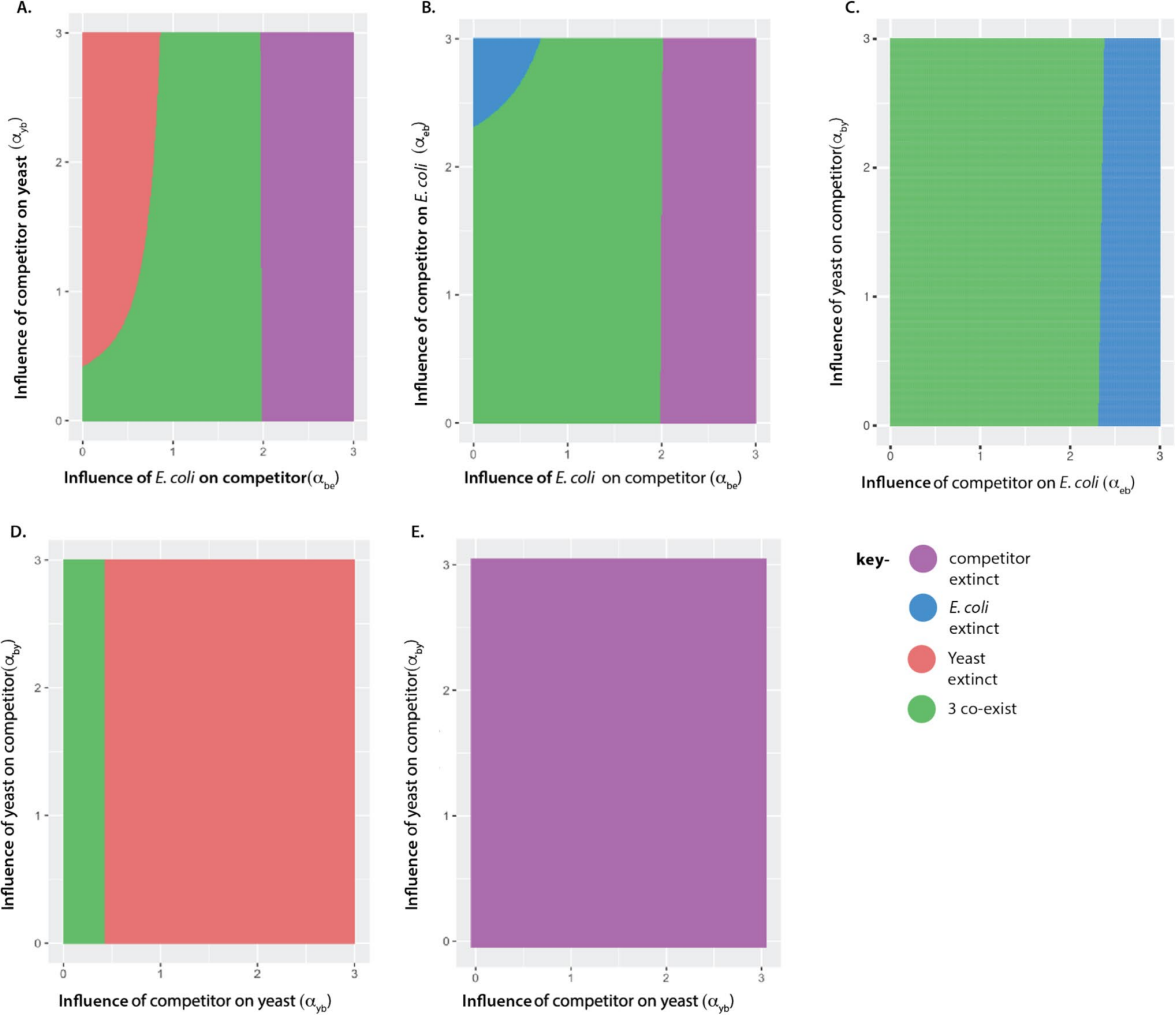
Simulations with a three-species Lotka-Volterra model. Each panel shows the outcomes of three species interactions for a range of alpha values after 24 h of growth following altered classic Lotka-Volterra (LV) models (Supplementary Methods). Simulations were run using carrying capacity and growth rate values from the experiment. Outcomes of coexistence or exclusion are shown on the right of the figure. Purple indicates only the competitor is extinct, blue shows only *E. coli* is extinct, red shows only yeast is extinct, and green shows that all three species coexist. A, B, C, D) All interaction values are fixed at 0.01 (for example, the interaction coefficients between *E. coli* and yeast) except for those on the x and y axes. A) Competition outcomes for a range of interaction coefficients; the influence of *E. coli* on the competitor (x-axis) and the influence of the competitor on yeast (y-axis). For example, yeast goes extinct even when the influence of the competitor is very small, but the competitor goes extinct when the interaction with *E. coli* is largely negative. B) Competition outcomes for a range of interaction coefficients; the influence of the competitor on *E. coli* (y-axis) and the influence of *E. coli* on the competitor (x-axis). C) Competition outcomes for a range of interaction coefficients; the influence of the competitor on *E. coli* (x-axis) and the influence of yeast on the competitor. D) Competition outcomes for a range of interaction coefficients; the influence of the competitor on yeast (x-axis) and the influence of yeast on the competitor (y-axis), with an interaction of *E. coli* on the competitor of 0.01. E) The same interactions between Yeast and the competitor are shown, but with an interaction of *E. coli* on the competitor of 2

To test whether our observations could be explained by three-way population dynamics, we extended a three-species Lotka-Volterra competition model to include frequency-dependent interactions for *E. coli* and yeast (Supplementary Methods). Using experimental data, we parameterized the model with growth rate (r) and carrying capacity (K) derived from our growth measurements, Fig. 2B, Supplementary Methods). We then simulated the outcomes of two- and three-species cultures, using *r* and K values taken from our empirical growth assays, and varied the between-species interaction coefficients to explore the conditions for coexistence (Fig. 4). The model includes low interaction coefficients for *E. coli* and yeast, which have been previously shown using a two-species Lotka-Volterra model (Fig. 2B) [7]. We incorporated a third species to investigate how increasing the competitive strength of the introduced competitor strain on yeast (α_yb_) influenced outcomes (Fig. 4A–D). We found that a higher competitive effect of the competitor strain on yeast increased the likelihood of yeast exclusion, but only when the impact of *E. coli* on the competitor (α_be_) was weak (Fig. 4A,D). When α_be_ exceeded 1, *E. coli* protected yeast from extirpation, and when α_be_ surpassed 2, the competitor strain was rapidly excluded from the co-culture (Fig. 4B,E).

## Discussion

Many studies of synthetic communities have focused on interactions between species that have been brought together independently of their evolutionary history [8, 11, 13, 18, 28, 33, 38]. A consistent finding of these studies has been that the results of two-way interactions between species can predict the outcome of three-way co-cultures [8, 11, 33]. Here we incorporated *E. coli*-yeast strains that had recently co-evolved for up to 4000 generations and found that *E. coli*-yeast pairs that are better adapted to the growth conditions of this experiment were more difficult to invade. In some cases, evolutionary history reduced the reliability of predictions made using two-species interactions – while yeast was outcompeted by some of the competitor bacteria in two-way co-cultures, this outcome was reversed when yeast’s coevolutionary partner *E. coli* was present. These results support that predictions about the function and composition of microbial communities will be influenced by the degree of adaptation to local conditions—including other species.

### Coevolution and Resistance to Invasion

The degree of adaptation to a local environment can have nuanced effects on the resistance to invasion. If a resident community has high productivity, this indicates that is well suited to extracting resources from local conditions, as supported by a screen of 680 microbial assemblages where invasion resistance was primarily predicted by whole-community productivity [25]. Although the abundance of one highly productive functional group (Enterobacterales) correlated with reduced invader success, modelling supported that this effect emerged from community-wide resource depletion rather than specific interactions with invading species [25]. In the case of a synthetic four-species consortium grown in a toxic environment, the resident species detoxified the medium, facilitating the invasion of competitor species [36]. However, after 44 weeks of coevolution in that environment that consortium effectively excluded new invaders. Dropout experiments demonstrated that no single member of the four-species group was indispensable. Instead, the authors attributed the heightened invasion resistance of the evolved community to priority effects and more comprehensive niche coverage developed during coevolution[36]. In this study, we did not explicitly test the importance of priority (or propagule) effects, as we consistently allowed our *E. coli*–yeast community time to acclimate before introducing competitor species, and didn’t vary the relative inoculation densities (Supplementary Methods). Nonetheless, our findings build on previous work by clearly demonstrating a species-level protective effect, where evolved *E. coli* protects yeast from — relative to yeast — competitively superior invaders. Interestingly, the only strain that could drive our 4000-generation evolved *E. coli* extinct was another strain of *E. coli* EAECO42 (Fig. 3C), supporting that, even in coevolved communities, strains may be most susceptible to an invader from the same species. Overall, these results support that as a species become better adapted to other species in their environment, and other abiotic environmental factors, that species will be better able to exclude less well adapted invading species.

### Resource Overlap Shields Yeast from Competitors in Three-Species Cultures

How can *E. coli* “protect” the relatively low abundance and slow-growing yeast cells from being displaced by competitively superior invading strains? This result aligns with the observations made about resource overlap. Our spent media assays demonstrated that *E. coli* and yeast draw on a distinct set of resources (Fig. 2BC), with yeast evolving to grow just as well on *E. coli* spent media as unspent media. This supports that during the 4000 generations of coevolution yeast has evolved reduced interactions with *E. coli*, corresponding to a small interaction coefficient (α_YE_) in the model. Conversely, the spent media assays show that the invader strains grow very poorly in *E. coli* and yeast spent media, suggesting that their growth will put them in direct competition with *E. coli* and yeast*,* reflected as the strong interaction coefficients. The strong negative effect of *E. coli* on the invading strain consequently diminishes the negative effect that the invading strain has on yeast, allowing for the maintenance of yeast. *E. coli*’s direct competition with the competitor is sufficiently intense so that the competitor is excluded before it can significantly reduce yeast’s population. These observations align with earlier work using Lotka–Volterra or resource competition models [11, 18, 30], to understand multispecies dynamics.

In a Lotka–Volterra framework, differences in resource use can be embedded directly into the pairwise interaction coefficients, ensuring that species with minimal resource overlap have only weak effects on each other. Additionally, the relative population sizes of the species shape their competitive influence: a species with tenfold greater population size can impose a much stronger effect than one with fewer cells. By integrating population size and resource overlap, we can better understand how pairwise interactions explain the dynamics of three-species systems. This approach provides a practical framework for extending two-species data into more complex ecological contexts.

## Conclusion

A major goal of microbial ecology is the deliberate assembly of communities of microbes with a targeted function. Together with findings from other large-scale studies of synthetic microbial communities, our results suggest that straightforward interaction data, leavened with an understanding of how interactions are modified by key functional traits such as resource preference, could be used to compose communities. However, using these data to predict the outcomes of three-species co-cultures is easier than predicting coexistence in complex multispecies assemblages [11, 49]. Our results support that a promising path to composing successful synthetic communities is directed evolution: Passaging communities in the actual conditions in which the community is expected to flourish, leads to synthetic consortia better equipped to resist invasion and persist over time.

## Supplementary Information

Below is the link to the electronic supplementary material.

Supplementary file1 (PDF 631 KB)

Supplementary file2 (DOCX 704 KB)

## Data Availability

Source data are provided as raw sequencing reads and FACS data and have been deposited GenBank under the Bioproject ID: PRJNA1023613.
