# Supplementary material for: Evolution of One Species Increases Resistance to Invasion in a Simple Synthetic Community
Source: Microb Ecol. 2025 Oct 20;88:110. doi: 10.1007/s00248-025-02618-w (PMC12537770; doi:10.1007/s00248-025-02618-w)
Supplement: Supplementary file 1 [file 248_2025_2618_MOESM1_ESM.pdf]

Supplementary Methods

1. **Making predictions about two-way interactions using growth data of species in monoculture (Fig. I-VII).**
2. **Parameterization of the generalized Lotka-Volterra model (Fig. VIII).**

1. Making predictions about two-way interactions using growth data.

We used monoculture growth measurements to make *a priori* predictions about the outcomes of two-species competition assays before they were performed. For each competitor–reference pairing (reference = *E. coli* or *S. cerevisiae*), we calculated three relative growth metrics:

1. **Relative growth rate (r)** – competitor r divided by the monoculture r of the reference strain.
2. **Relative carrying capacity (K)** – competitor K divided by the monoculture K of the reference strain.
3. **Relative area under the logistic growth curve (AUC_l)** – competitor AUC_l divided by the monoculture AUC_l of the reference strain.

For each metric, we determined an arbitrary "coexistence range" defined as ±1 standard deviation around the mean relative value for the reference organism (which is 1.0 by definition). This resulted in cut-offs of 0.85–1.15 for growth rate and 0.90–1.10 for both K and AUC_l. Competitors with relative values within the coexistence range were predicted to coexist with the reference organism. Values below the lower cut-off were predicted to result in exclusion of the competitor by the reference, and values above the upper cut-off were predicted to result in exclusion of the reference by the competitor. This broader coexistence range is based on the observation that, although very large differences in monoculture growth rate can lead to competitive exclusion, a wide range of intermediate growth-rate differences can still result in stable coexistence (Friedman et al 2017). In addition, we developed this "cut-off" approach because while previous work, assessed monoculture growth rates *post hoc* for their ability to explain observed competition outcomes, we aimed to generate predictions before running the empirical competition assays.

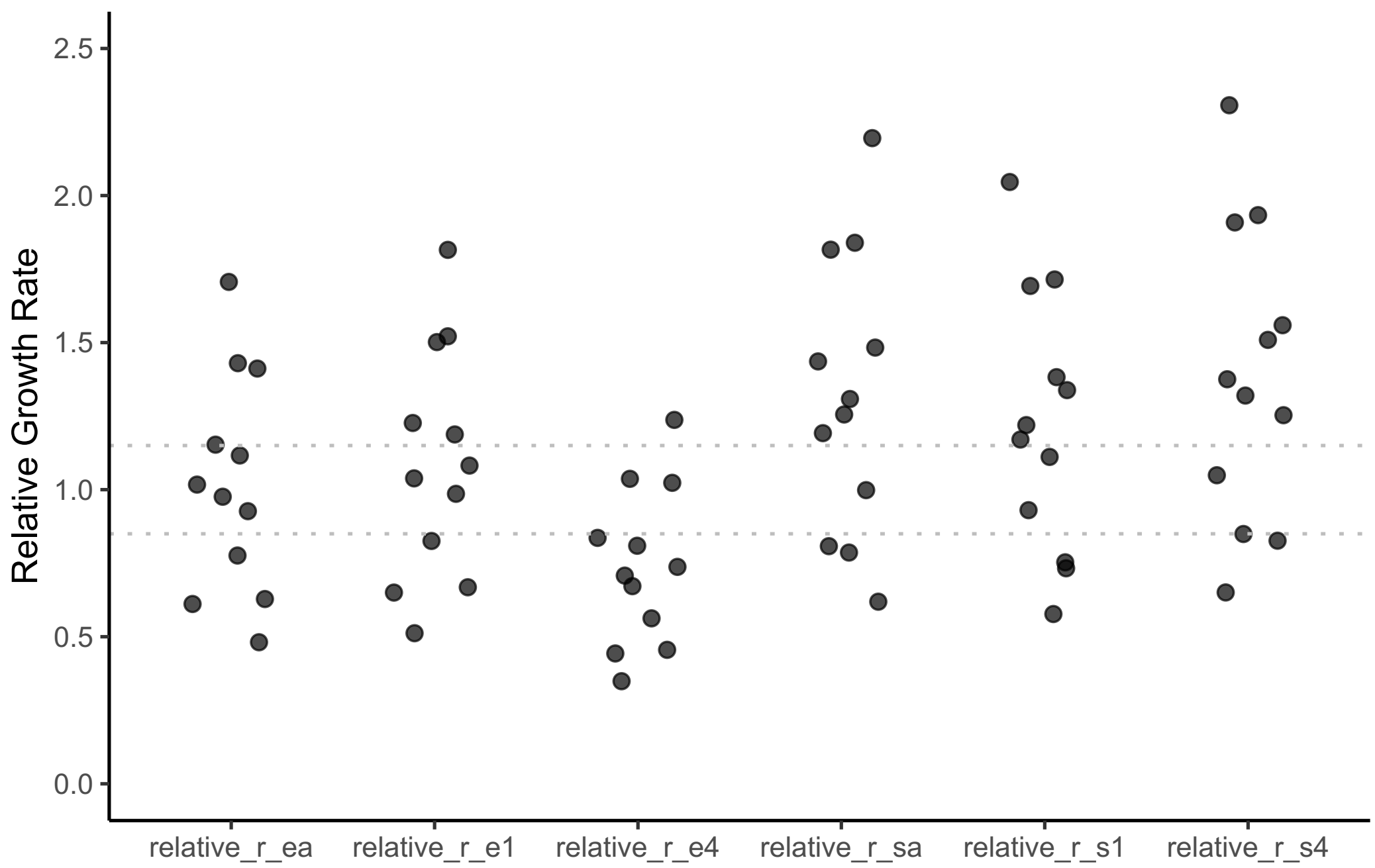

Figure I: Relative growth rate of competitor bacteria to *E. coli* or yeast in monoculture. Relative growth rates (y-axis) were calculated as the ratio of a competitor bacteria's growth rate (r) to that of *E. coli* or yeast when grown in monoculture. Ancestral, 1000-generation evolved, and 4000-generation evolved yeast (sa, s1 and s4) or *E. coli* (ea, e1, e4) with competitors are shown on the x-axis. X-axis abbreviations: ea indicates ancestral *E. coli*, e1 1000 generation evolved *E.* coli, e4 4000 generation evolved *E.* coli, sa ancestral *S. cerevisiae* (yeast), s1 1000 generation evolved *S. cerevisiae* (yeast), s4 4000 generation evolved *S. cerevisiae* (yeast). The range of coexistence falls between relative growth rate values of 0.85–1.15 (indicated by dashed lines), which were determined based on the standard deviation of relative growth rates in yeast and *E. coli*. Competitive exclusion is expected when a competitor's relative growth rate falls outside the coexistence range: competitors with a relative r < 0.85 are predicted to be excluded by yeast or *E. coli*, while those with a relative r > 1.15 are expected to exclude yeast or *E. coli*.

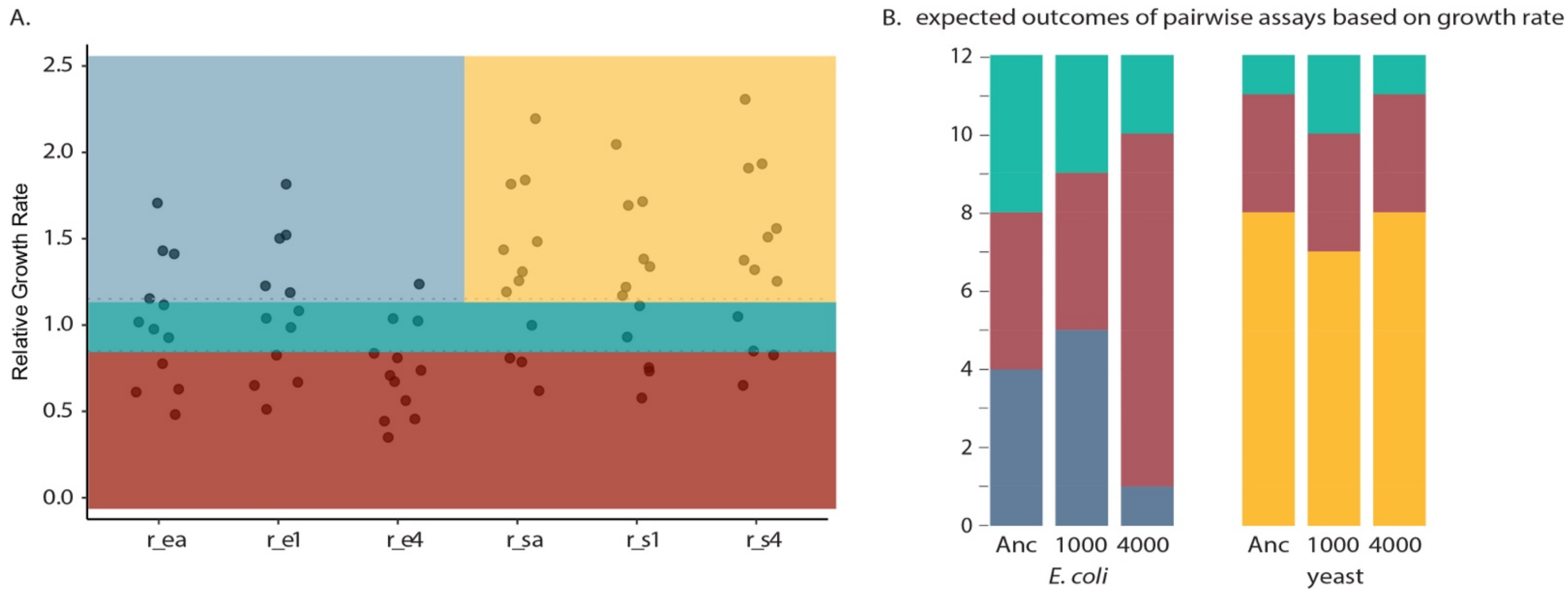

Figure II: Predicted two-way competition outcomes based on growth rate (data used to generate main text Fig. 3**D**)**.** Allocation of expected competition outcomes. Panel **A**) Relative growth rates that fall within the green area indicate pairs of strains that were expected to coexist. Red shows pairs where the competitor strain is expected to be excluded. Blue shows pairs where *E. coli* is expected to be outcompeted, and yellow where yeast was expected to be excluded based on growth rates. Relative growth rates (y-axis) were calculated as the ratio of a competitor bacteria's growth rate (r) to that of yeast or *E. coli* when grown in monoculture. Ancestral, 1000-generation evolved, and 4000-generation evolved yeast or E. coli with competitors are shown on the x-axis. X-axis abbreviations: ea indicates ancestral E. coli, e1 1000 generation evolved *E. coli*, e4 4000 generation evolved *E. coli*, sa ancestral *S. cerevisiae* (yeast), s1 1000 generation evolved *S. cerevisiae* (yeast), s4 4000 generation evolved *S. cerevisiae* (yeast). The range of coexistence falls between relative growth rate values of between 0.85–1.15 (shown as dashed lines), which were determined based on the standard deviation of relative growth rates in yeast and *E. coli.* Competitive exclusion is expected when a competitor's relative growth rate falls outside the coexistence range: competitors with a relative r < 0.85 are predicted to be excluded by yeast *or E. coli,* while those with a relative r > 1.15 are expected to exclude yeast or *E. coli*. Panel **B**) indicates expected outcomes of competition shown in panel A as a stacked bar chart.

In addition to using growth rates, we used relative carrying capacity (K, Fig. **IIIA**) and "area under the curve" (AUC, Fig. **IIIB**) to make *a priori* predictions of two-species competitions of the bacteria with either *E. coli* or yeast.

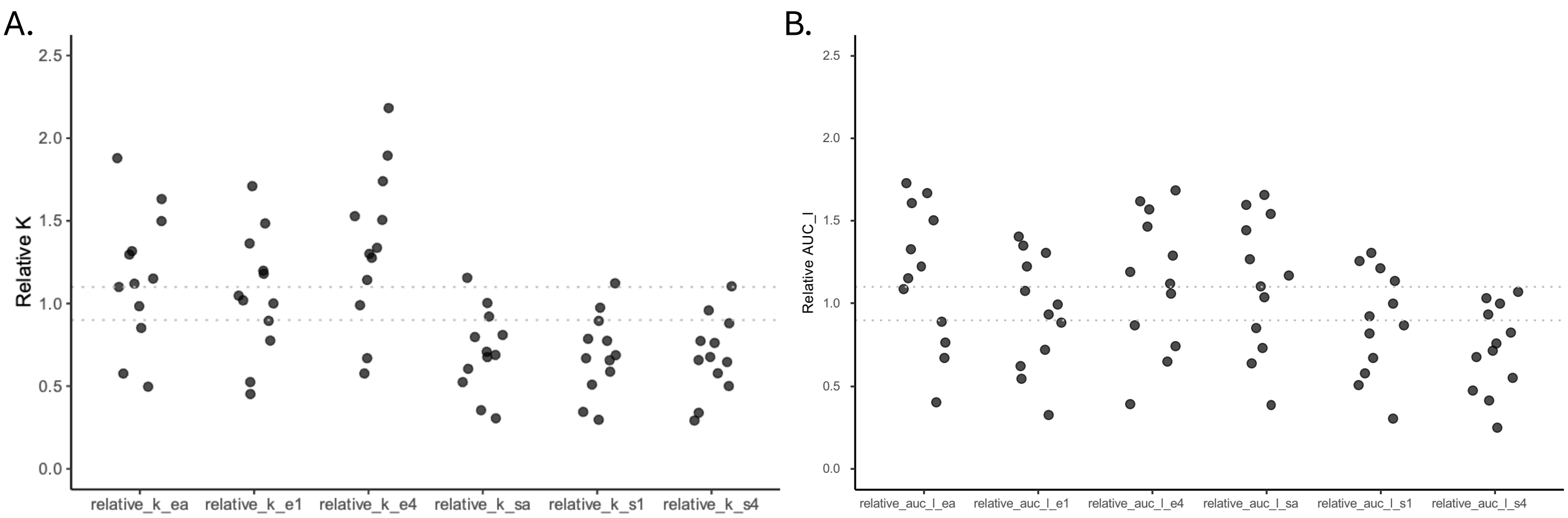

Figure III: Expected competition outcomes between competitor bacteria and yeast or E. coli based on relative carrying capacity (A) and relative logistic area under the curve (AUC_l) (B). Relative values were calculated by dividing either the K or AUC of the competitor by the K or AUC of either yeast or *E. coli*. Competitors with relative values of below 0.9 are expected to be excluded by either yeast or *E. coli*, and competitors with relative values within a range of 0.9-1.10 are expected to coexist whereas competitors with relative values above 1.10 are expected to exclude either yeast or *E. coli.*

We found that growth rate does not predict the dominant species in pairwise competitions (Fig. **IV**). To determine if outcomes match predictions, we compared our predictions to the outcomes of two-way cocultures. When relative growth rate is greater than 1, and outside the "green" band of coexistence (Fig. **IIA**), the competitor is expected to dominate the focal yeast or *E. coli* strain. Where relative growth rate is less than 1, the competitor is expected to be outcompeted. We assessed prediction accuracy by the number of outcomes where outcomes matched predictions. Almost all growth rate characteristics had a prediction accuracy of less than 50% for all two-way species competition outcomes. Only the growth rate of 4000-generation evolved *E. coli* had a high prediction accuracy of >75%, as this was often the fastest-growing species and resulted in the greatest number of competitor exclusions. Prediction accuracy was only greater than 70% for two-way competitions between competitors and ancestral *E. coli* (prediction accuracy 72.7%) and 4000-generation evolved *E. coli* (prediction accuracy 83.33%).

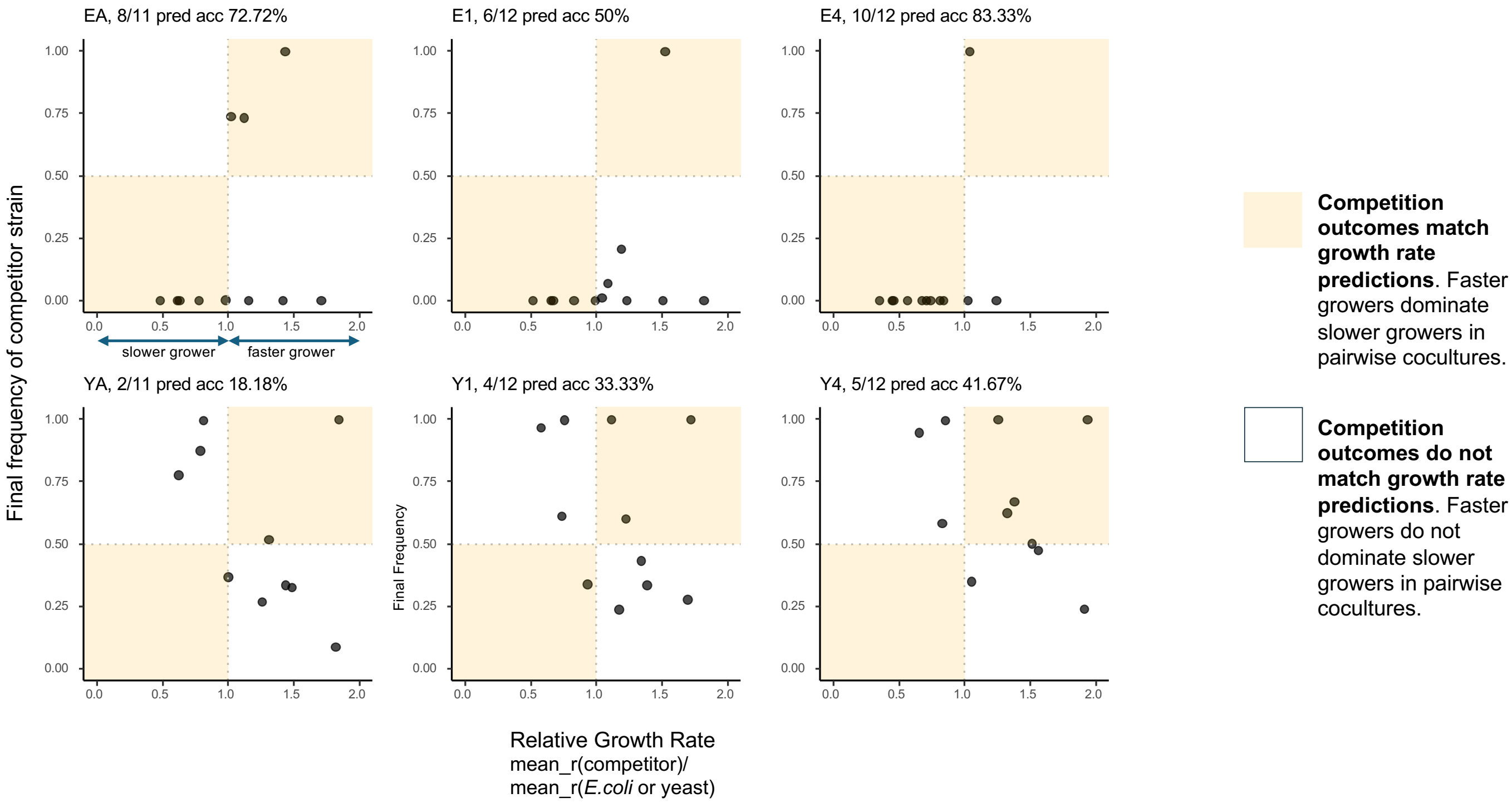

Figure IV: Number of times the outcomes matched the predictions of two-way competition outcomes predicted by relative growth rate. The y-axes show relative final frequency of the bacterial strain in competition with either *E. coli* or yeast after 7 days of coculture. X-axes show relative growth rate values, calculated as the mean growth rate of the competitor bacteria divided by the mean growth rate of either *E. coli* or yeast (from Fig. II). Each dot shows one of the 12 bacterial competitors. Yellow zones show cases where outcomes matched growth rate predictions, and white squares show where outcomes did not match predictions.

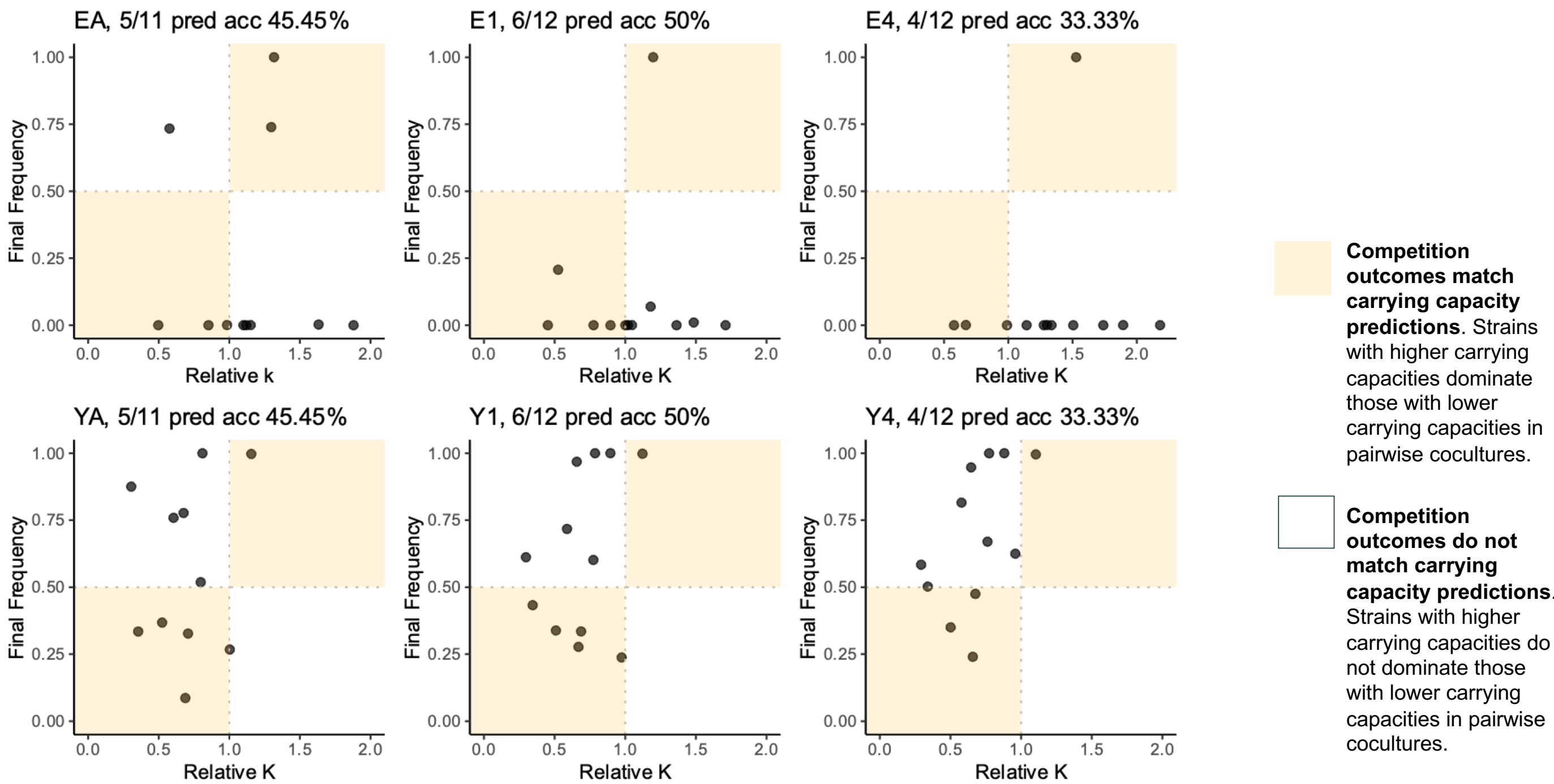

Figure V: Number of times the outcomes matched the predictions of two-way competition outcomes predicted by relative carrying capacity. The y-axes show relative final frequency of the bacterial strain in competition with either *E. coli* or yeast after 7 days of coculture. X-axes show relative carrying capacities (K), calculated as the mean carrying capacity of the competitor bacteria divided by the mean carrying capacity of either *E. coli* or yeast. Each dot shows one of the 12 bacterial competitors. Yellow zones show cases where outcomes matched carrying capacity predictions, and white squares show where outcomes did not match predictions.

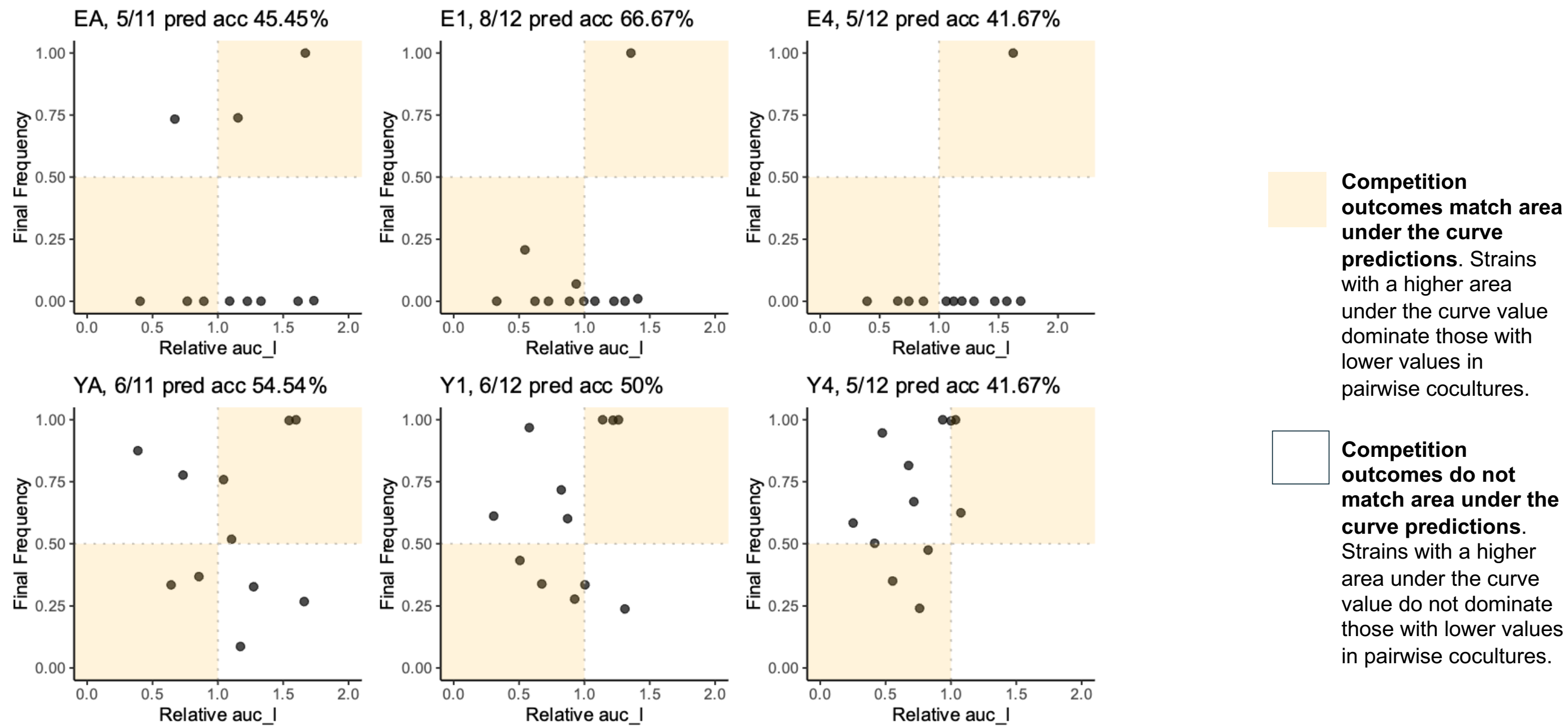

Figure VI: Number of times the outcomes matched the predictions of two-way competition outcomes predicted by relative area under the curve. The y-axes show relative final frequency of the bacterial strain in competition with either *E. coli* or yeast. X-axes show relative area under the curve values, calculated as the mean area under the curve of the competitor bacteria divided by the mean area under the curve of either *E. coli* or yeast. Each dot shows one of the 12 bacterial competitors. Yellow zones show cases where outcomes matched area under the curve predictions, and white squares show where outcomes did not match predictions.

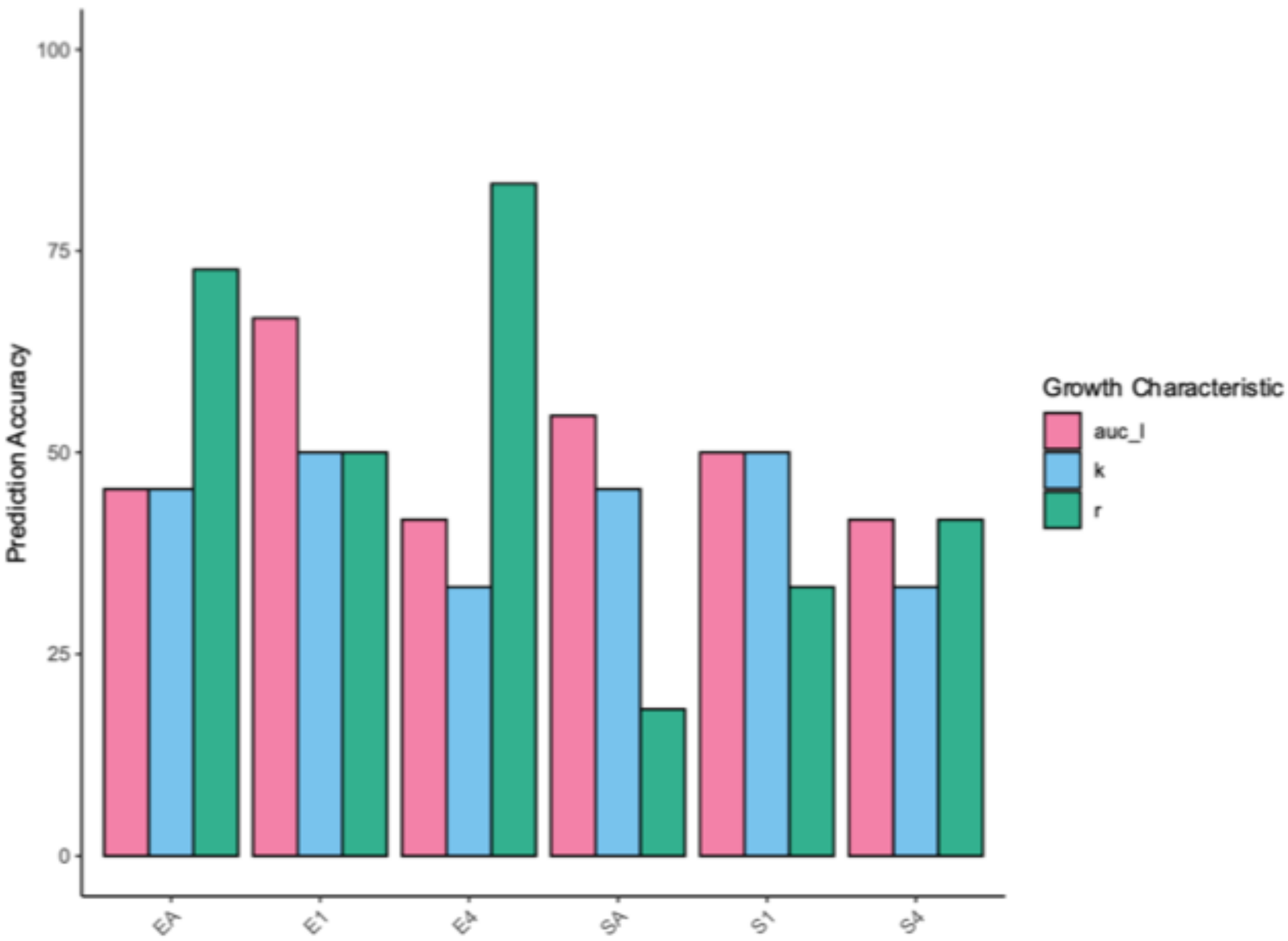

Figure VII: Prediction accuracy of growth characteristics. The accuracy of growth rate (r), carrying capacity (K) and area under the curve (AUC_l) in predicting empirical two-species competition outcomes. Groups on the x-axis show predictions between competitor bacteria and ancestral *E. coli* (EA), 1000-generation evolved *E. coli* (E1), 4000-generation evolved *E. coli* (E4), ancestral yeast (YA), 1000-generation evolved yeast (1), 4000-generation evolved yeast (Y4).

2. Parameterization of the generalized Lotka-Volterra model

Here we explain how the model was parameterised and used in relation to the empirical data generated in the study. Using this model, we found that a larger negative interaction between *E. coli* and the competitor could counteract the negative interaction between yeast and the competitor in 3-species cocultures.

To make the model, we modified the classic Lotka-Volterra model to include frequency dependence and interactions between 3 species. To test whether the model could realistically depict biological outcomes, we ran simulations with a range of carrying capacities, growth rates and interaction coefficients (alphas). All growth rates and carrying capacities were based on empirical measurement and are shown in Sup. Fig. 2. The interaction coefficients were not based on empirical data. The twelve competitor species used in the experiment have a range of carrying capacities and growth rates either above, below or equal to either yeast or *E. coli*. We found that varying carrying capacity and growth rate of competitors, *E. coli* or yeast, did not result in competitive exclusion if interaction coefficients were small. We show some simulations (Fig. VIII), noting that changing to other values of carrying capacity or growth rate still resulted in coexistence. To test the importance of interaction coefficients in predicting competition outcomes, we modelled the range of interaction coefficients which resulted in either coexistence or exclusion in 3-species communities (Fig. 4**A-E**, main text). Simulations varied only two interaction coefficients at a time, while all other interaction coefficients were at a constant low value. We observed coexistence when interaction coefficients were small, and competitive exclusion as interaction coefficient values increased.

Parameters	Unequal growth rates and carrying capacities and equal interaction coefficients.	Unequal growth rates, equal carrying capacities and equal interaction coefficients.	Equal growth rates, carrying capacities and interaction coefficients.
Parameter values	K.e=K.b=1*10^8, K.y=1*10^7; r.e=0.8; r.y=0.5; r.b=0.6; allalphas= 0.01	K.e=K.b=1*10^8 K.y=1*10^7; r.e=r.b=0.6, r.y=0.5; all alphas= 0.01	K.b=K.e=K.y=1*10^8; r.e=r.b=r.y=0.6; all alphas= 0.01
Simulation outcomes	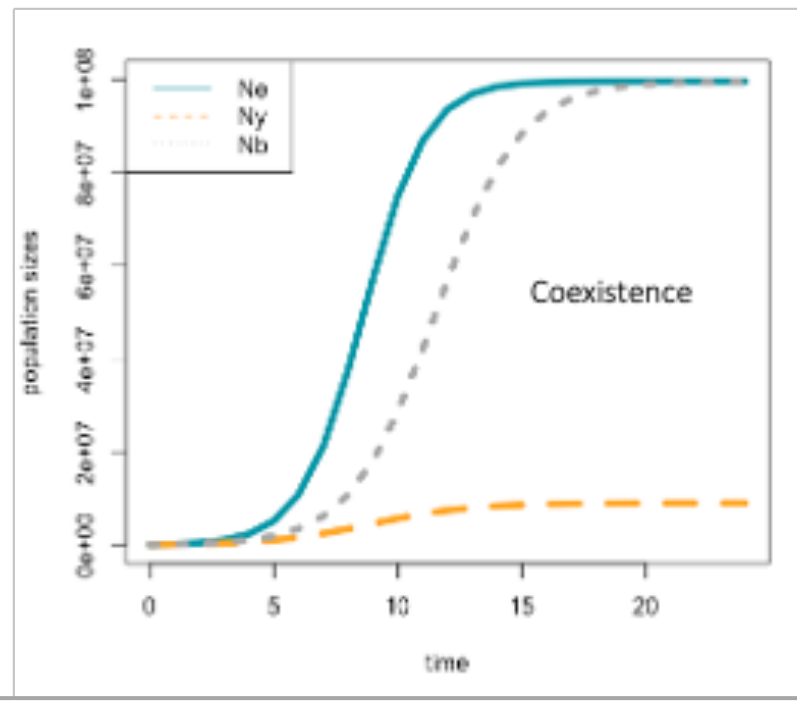	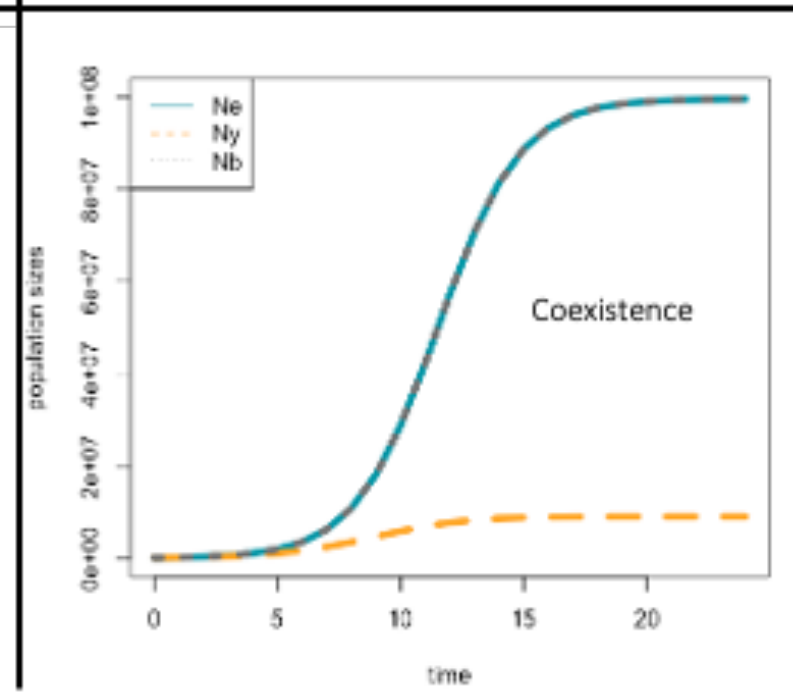	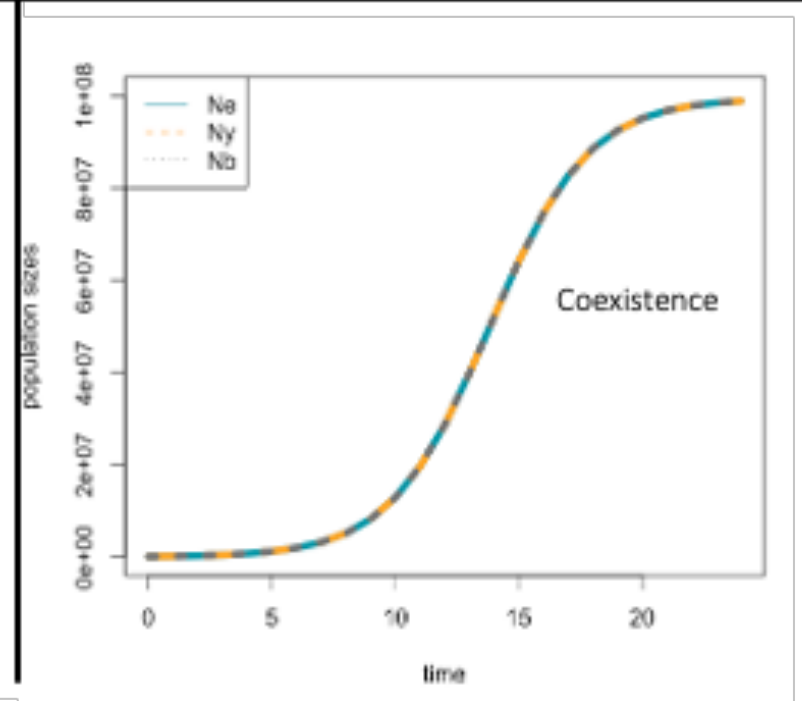

Figure VIII: Simulation outcomes using the modified 3-species Lotka-Volterra model. Parameters and parameter values used to run the simulations are shown in the first two rows above. Simulation outcomes are shown as population sizes (y-axis) of the 3-species cocultures over time (x-axis). Dashed yellow lines are yeast, blue lines are *E. coli* and grey lines are competitor bacteria. Simulations were run in R v 4.2.2.
