# Supplementary material for: Evolution of One Species Increases Resistance to Invasion in a Simple Synthetic Community
Source: Microb Ecol. 2025 Oct 20;88(1):110. doi: 10.1007/s00248-025-02618-w (PMC12537770; doi:10.1007/s00248-025-02618-w)

**Supplementary Figures**


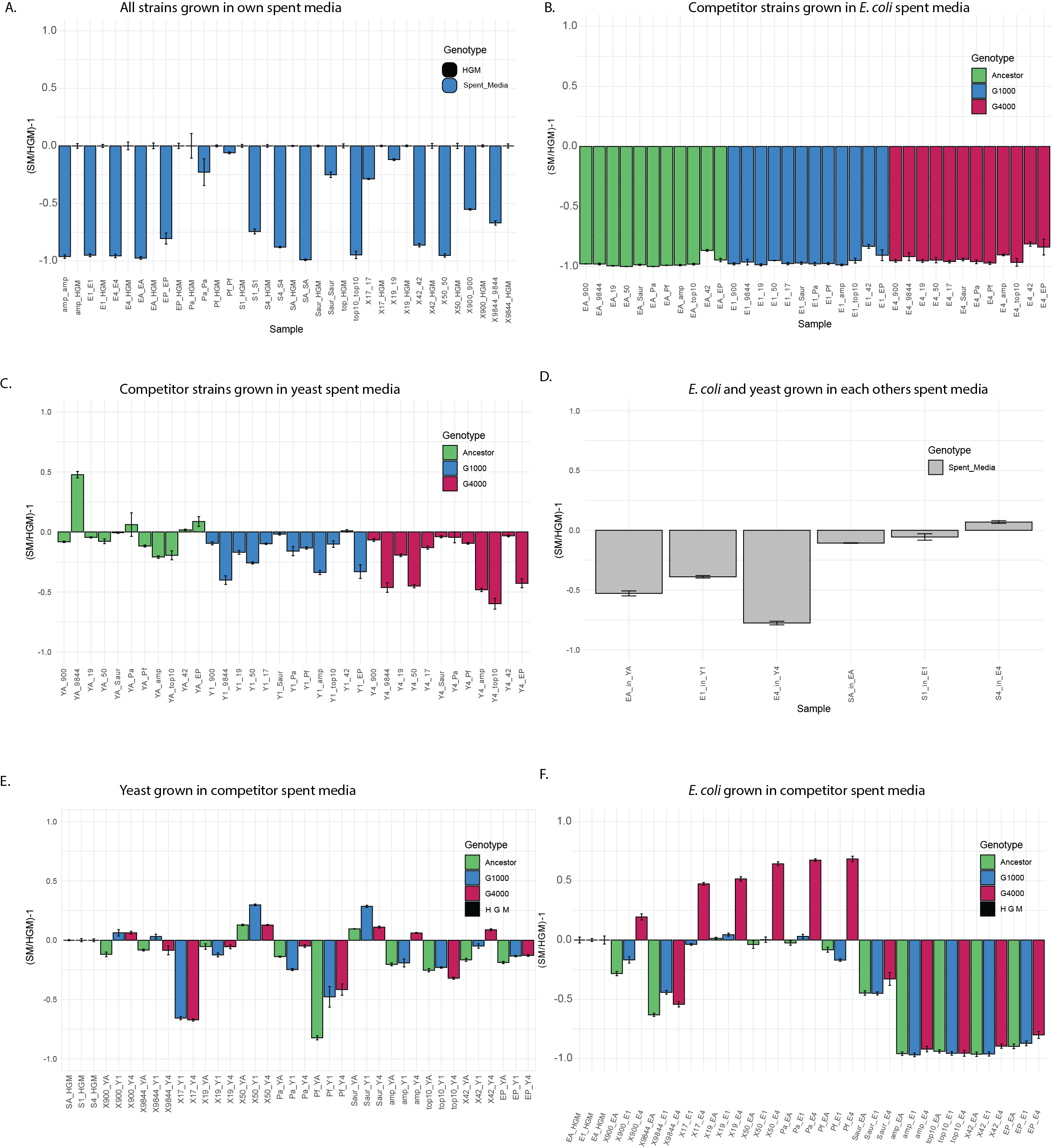


**Supplementary Figure 1:** Spent media assay results, shown as spent media carrying capacity (SM) divided by high-glucose media (HGM) carrying capacity, minus 1. A value of -1 indicates no growth, 0 shows equal growth in high glucose media (HGM) and a positive value shows enhanced growth in spent media (SM). A) Each strain, including competitors, yeast, and E. coli, grown in its own spent media. B) Competitors grown in E. coli spent media, where ancestral strains are in green, G1000 in blue and G4000 in red. C) Competitors grown in yeast spent media, where ancestral strains are in green, G1000 in blue and G4000 in red. D) E. coli and yeast grown in each other’s spent media. E) Yeast grown in competitor spent media, where ancestral strains are in green, G1000 in blue and G4000 in red. F) E. coli grown in competitor spent media, where ancestral strains are in green, G1000 in blue and G4000 in red. Statistical significance was determined using either a one-way or two-way analysis of variance (ANOVA).


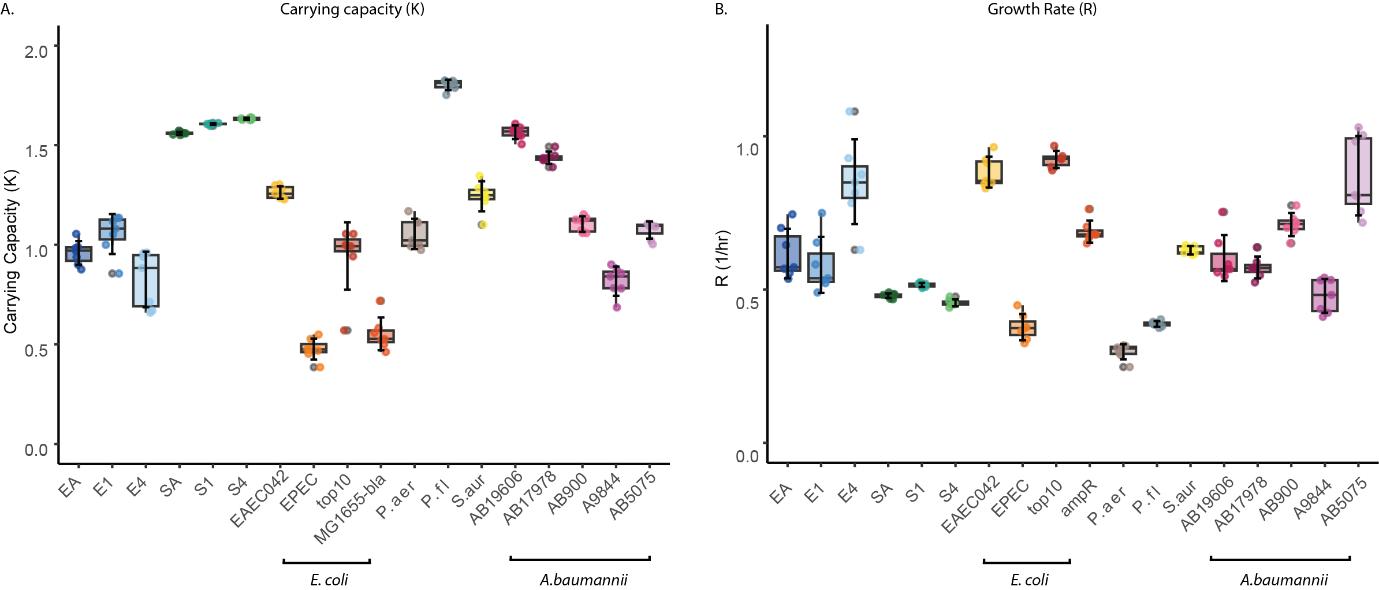


**Supplementary Figure 2**. A) Carrying capacity (K) and B) growth rate for each species in monoculture using growth curves carried out over 24hours in the conditions of the evolution experiment. Each species and strain reached carrying capacity in HGM within 24 hours. Error bars indicate standard deviation (Methods). Carrying capacity calculated as maximum population size for each species is as follows shown as the number of Colony Forming Units per milliliter of culture (CFU/ml): EA=4.4x10^8^, E1=4.2x10^8^, E4=3.2x10^8^, SA=7.3x10^7^, S1=2.8x10^7^, S4=5x10^7^, EAEC042=6.6x10^8^, EPEC=3.5x10^8^, top10=7x10^8^, MG1655-bla=2.8x10^8^, P. aeruginosa=1.1x10^8^, P. fluorescens=5x10^8^, S. aureus=5.7x10^8^, AB19606=7.8 x10^8^, AB17978=2.4x10^8^, AB900=6.9x10^8^, A9844=1.9x10^8^ and AB5075=4.3x10^8^.


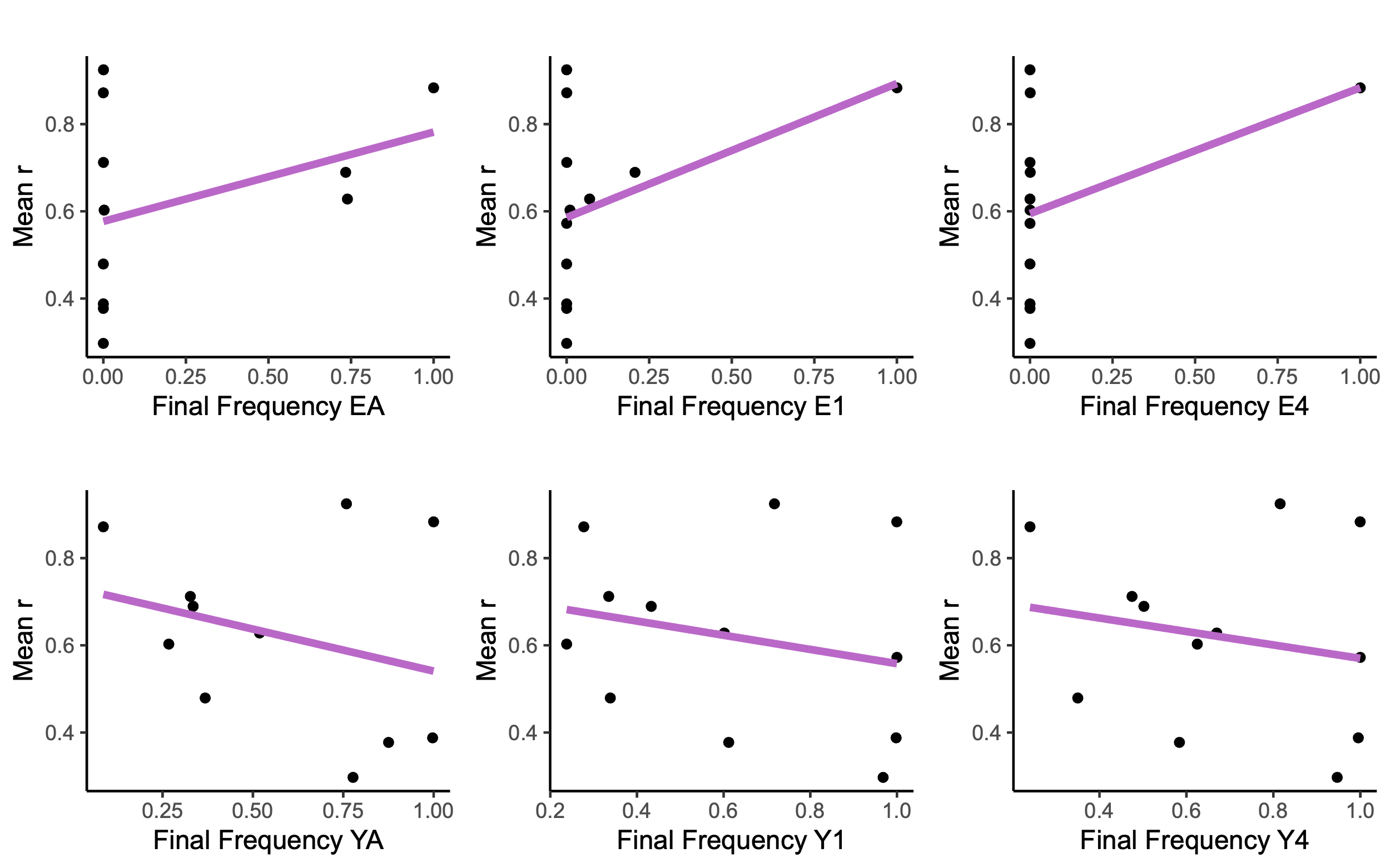


**Supplementary Figure 3. Relationship between growth rate and the final frequency after 7 days of co-culture with *E. coli* or yeast.** Each panel represents a different *E. coli* or yeast strain (A = ancestor, 1 = 1000 generation evolved, and 4 = 4000 generation evolved. Final frequency of the competitor bacteria strain after 7 days of co-culture plotted on the x-axis. Mean growth rate (r) of the bacterial competitor strain is plotted on the y-axis. Black dots indicate a different competitor bacterial species, and the purple line represents a linear model fit. Normality of the data was assessed using Q-Q plots, histograms, and the Shapiro-Wilk test in R. Since final frequency was non-parametric, Spearman’s rank correlation test was performed, revealing no significant correlation in any comparison.

**Supplementary Figure 4. Example gating for flow cytometry.**


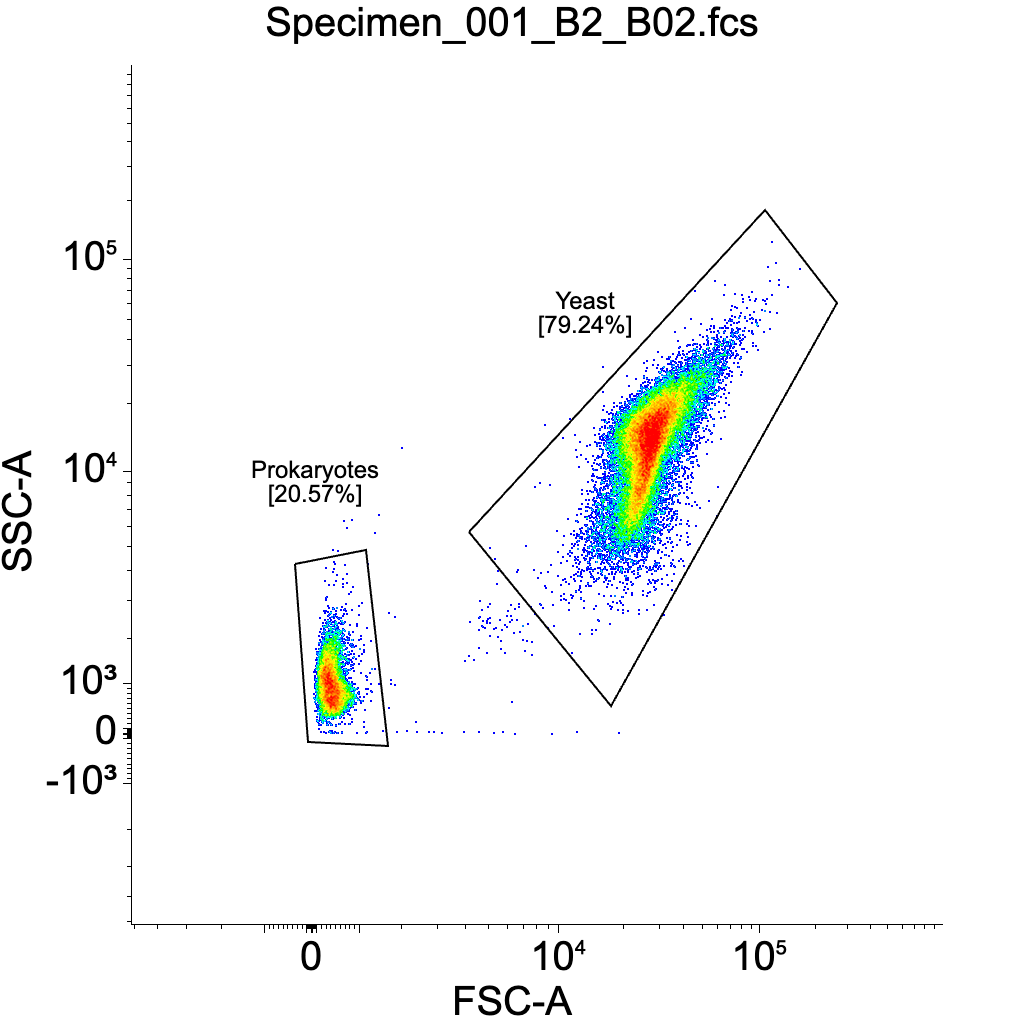

Supplement: Supplementary file 2 — Supplementary file2 (DOCX 704 KB) [file 248_2025_2618_MOESM2_ESM.docx]
